# Lifetime risk of incident dementia and incident mild cognitive impairment in older adults

**DOI:** 10.1002/alz.71173

**Published:** 2026-02-03

**Authors:** Lianlian Du, Lei Yu, Tianhao Wang, Patricia A. Boyle, Lisa L. Barnes, David X. Marquez, David A. Bennett

**Affiliations:** ^1^ Rush Alzheimer's Disease Center Rush University Medical Center Chicago Illinois USA; ^2^ Department of Neurological Sciences Rush University Medical Center Chicago Illinois USA; ^3^ Department of Psychiatry and Behavioral Sciences Rush University Medical Center Chicago Illinois USA; ^4^ Department of Kinesiology and Nutrition University of Illinois Chicago Chicago Illinois USA

**Keywords:** dementia, lifetime risk, mild cognitive impairment, race and ethnicity, sex

## Abstract

**INTRODUCTION:**

We estimated the lifetime risk of incident dementia and mild cognitive impairment (MCI) from ages 55–105 and examined differences by sex and race.

**METHODS:**

Data were drawn from five harmonized longitudinal cohort studies at the Rush Alzheimer's Disease Center, including 4611 participants for dementia and 3915 for MCI. Diagnoses were based on annual clinical evaluations. Lifetime risk was estimated using nonparametric cumulative incidence curves by age conditional on being alive and event‐free at the index age, accounting for competing mortality and delayed study entry, stratified by sex and race.

**RESULTS:**

Lifetime risk from age 55 was 43% (95% confidence interval [CI]: 38%–47%) for dementia and 62% (95% CI: 57%–67%) for MCI. Female participants had higher risks than male participants, and racial differences were modest.

**DISCUSSION:**

These findings extend lifetime risk estimation beyond age 90 among diverse older adults and provide MCI estimates, emphasizing equity‐focused prevention and public health strategies to reduce cognitive impairment.

**Highlights:**

Lifetime risk (cumulative incidence) of dementia and mild cognitive impairment (MCI) was estimated from ages 55 to 105 using nonparametric cumulative incidence models accounting for competing risk of death and left truncation.

The estimated lifetime risk was 43% for incident dementia and 62% for MCI, with risk rising steeply after age 75 and appearing to level off at the oldest ages.

Women and Black participants showed higher lifetime risks, partly reflecting mortality and selective survival dynamics.

Exploratory analyses suggested elevated risks among Latino participants and those with a history of stroke.

These findings extend lifetime risk estimation beyond age 90 and highlight the need for equitable, culturally informed dementia prevention and monitoring strategies.

## 1 BACKGROUND

Dementia is a growing public health crisis, affecting over 7 million Americans and projected to reach about 14 million by 2060.^1^, ^2^ Among individuals 90–95 years of age, it contributes to over 40% of deaths among female participants and 30% among male participants.^3^ The financial impact is severe, with health care, long‐term care, and unpaid caregiving costs projected to exceed $700 billion in 2024.^2^ Mild cognitive impairment (MCI) an intermediate clinical stage associated with elevated risks of dementia and mortality, affects more than 12 million individuals in the United States as of 2020, and is expected to increase to 22 million by 2060.^1^ Prevalence increases with age, from 7% at 60–64 to 25% at 80–84.^4^ Since no cure exists for dementia, prevention and early risk stratification are critical.^5^

Lifetime dementia risk is a critical public health metric that informs policy, supports stakeholder engagement (e.g., American Heart Association, Alzheimer's Association), and helps estimate the potential benefits of early interventions.^6^, ^7^, ^8^ However, accurate estimation is methodologically challenging due to competing mortality and left truncation from variable age at study entry. In prior work, we found that individuals diagnosed after age 85 survived less than 3 years on average,^9^ highlighting the need for short‐interval assessments in the oldest old to capture incident cases. Standard methods such as the Kaplan–Meier estimator overestimate risk by ignoring competing events, particularly problematic in late‐life diseases like dementia.^10^ Proper estimation must therefore incorporate both competing risks and delayed entry.^11^

Here, we employ a nonparametric cumulative incidence function based on the Aalen–Johansen estimator to directly estimate absolute risk under competing risks and accommodate left truncation when age is used as the time scale.^11^ Its influence–function (pseudo‐observation) representation provides a flexible framework for inference on cumulative incidence without relying on proportional subdistribution hazard assumptions required by Fine–Gray models.

Given known sex, racial, and ethnic disparities in dementia risk, subgroup‐specific lifetime risk estimates are essential.^6^, ^7^, ^12^ Prior research has made significant advances in documenting the risk and burden of cognitive impairment.^7^, ^13^, ^14^, ^15^, ^16^ However, few studies include large numbers of individuals age 90+, most studies focus on non‐Latino White populations,^12^ and only one study to date has estimated lifetime risk for MCI.^13^ A recent study using a subset of these data predicted dynamic lifetime risk of Alzheimer's disease using longitudinal cognitive assessment data,^17^ but did not estimate absolute lifetime risk accounting jointly for competing mortality and delayed entry. As cognitive impairment grows more prevalent in an aging, tech‐dependent society,^18^ efforts to refine and extend lifetime risk estimates are increasingly urgent.

Here, we used harmonized longitudinal data from five Rush Alzheimer's Disease Center (RADC) cohorts to estimate lifetime risks of incident dementia and incident MCI. We further examined differences by sex and race to characterize disparities in risk across aging populations.

## 2 METHODS

### 2.1 Participants

We used data from five harmonized longitudinal cohort studies at the RADC: Religious Orders Study (ROS),^19^ Rush Memory and Aging Project (MAP),^19^ Minority Aging Research Study (MARS),^20^ African American Clinical Core (AACORE),^21^ and the Latino CORE (LATC).^22^ All participants were community‐dwelling older adults without known dementia at enrollment and agreed to annual clinical evaluations with high retention (withdrawal rate <10%). These studies share similar protocols for design and data collection, as outlined previously.^22^ For lifetime dementia risk estimation, we excluded participants with dementia at baseline (*n* = 310); those without follow‐up data (*n* = 378), including individuals who had not yet reached the time window for their annual follow‐up evaluation as of December 2025; those who died, or those who declined further clinical participation, and participants younger than age 55 at baseline (*n* = 8) to support left truncation. To ensure diagnostic consistency, we further excluded 280 deceased participants whose last visit was >3 years before death (see Section 2.2), resulting in an analytic sample of 4677. For lifetime MCI risk estimation, we additionally excluded participants with MCI at baseline (*n* = 687), yielding a final sample of 3990. See Figure S1 (STrengthening the Reporting of OBservational studies in Epidemiology [STROBE] flow diagram) for details. All participants provided written informed consent; individual studies were approved by an institutional review board at Rush University Medical Center.

**RESEARCH In CONTEXT**

1. **Systematic Review**: We searched PubMed from database inception through December 2025 using the terms lifetime risk, cumulative incidence, dementia, and mild cognitive impairment (MCI), along with methodological terms related to competing risks (e.g., subdistribution, cumulative incidence function). Prior population studies often reported lifetime dementia risk at selected index ages, but reports for MCI were uncommon. Many estimates did not explicitly account for competing mortality or delayed study entry, which are critical in late‐life diseases. Few studies included substantial numbers of adults 90 years of age or older. Stratified estimates by sex and by race or ethnicity were also limited and methodologically heterogeneous, which reduced comparability.
2. **Interpretation**: Using data from five harmonized, community‐based cohorts from the Rush Alzheimer's Disease Center with annual clinical evaluations, we estimated lifetime risks of dementia (43%) and MCI (62%) from ages 55 to 105. We applied nonparametric cumulative incidence models that used age as the time scale, adjusted for delayed study entry, and accounted for death as a competing risk. Results are reported overall and stratified by sex and race, with exploratory analyses examining Latino ethnicity, socioeconomic status, and vascular risk factors.
3. **Future Directions**: Competing‐risk lifetime‐risk curves for dementia and MCI can inform service planning, caregiver and long‐term care capacity, and risk communication for clinicians, patients, and families. Stratified and exploratory estimates highlight population differences by sex, race, ethnicity, and vascular health that may guide targeted prevention and early intervention. Future studies should validate these findings in larger and more diverse populations and integrate biological, social, and behavioral factors to improve personalized lifetime risk prediction.

### 2.2 Dementia and MCI ascertainment

All five RADC cohorts follow a standardized diagnostic protocol.^22^, ^23^ At baseline and annual follow‐ups, participants undergo a structured evaluation including medical history, neurological examination, and neuropsychological testing. Diagnoses are determined via a validated three‐step process and validated across numerous RADC studies.^23^, ^24^, ^25^ Dementia diagnoses follow the National Institute of Neurological and Communicative Disorders and Stroke and the Alzheimer’s Disease and Related Disorders Association (NINCDS/ADRDA) criteria.^26^ MCI is defined as cognitive impairment per neuropsychologist review without meeting dementia criteria. Of note, clinicians were masked to previously assigned diagnostic classifications for all participants when conducting the clinical evaluation and assigning the current diagnosis, that is, each follow‐up evaluation was performed identically to the baseline evaluation. Diagnoses were determined using validated, multi‐step decision rules that included an assessment of the neuropsychological tests and an independent evaluation for diagnostic classification. In discrepant cases, the clinicians conferred to render a joint decision. A third clinician provided input in the rare instances when agreement could not be reached.

Dementia was treated as an absorbing state, with onset defined as the first diagnosis. For MCI, we used a conservative definition requiring persistence or progression. An initial MCI classification was considered incident if it was followed by a subsequent clinical evaluation classified as MCI or dementia, or if death occurred after the MCI visit when no further evaluation was available. Those transitioning from MCI to death had cognitive scores comparable to those remaining with MCI, supporting their inclusion (see Figure S2).

### 2.3 Demographic characteristics, comorbidities, and socioeconomic status (SES)

All participants self‐reported their sex (male or female), date of birth, and years of education. Age was calculated based on the time between the reported birth date and the date of clinical assessment. Race and ethnicity were self‐reported at enrollment using categories consistent with the 1990 U.S. Census format. Race was classified as White or Black for primary race‐stratified analyses, regardless of Latino ethnicity; participants from other racial groups were excluded from race‐specific analyses due to small sample sizes. Latino ethnicity (yes/no) was examined separately in exploratory analyses, given the limited numbers and younger age distributions.

**Vascular risk burden**.^27^ A composite score (range 0–3) was derived from self‐reported history of hypertension, diabetes, and smoking. Hypertension was considered present if reported at baseline or any follow‐up visit. Diabetes was coded as present based on self‐report or diabetes medication use. Smoking history was based on the baseline report and coded as present for current or former smokers.

**Vascular disease burden**.^28^ This index (range 0–3) captured history of claudication, stroke, and heart conditions. Claudication was based on reported calf pain during walking. Stroke included probable or highly probable diagnoses based on participant report or clinical evaluation. Heart conditions included myocardial infarction and related events reported at baseline or during follow‐up. The composite score was computed if at least two items were available.

**Socioeconomic status (SES)**. Early life socioeconomic status (SES) was assessed using a composite index based on three self‐reported indicators obtained at baseline: paternal education (years), maternal education (years), and number of children in the family. The number of children was multiplied by –1 so that higher values reflected higher SES. Each indicator was standardized, and the SES score was calculated as the mean of the three *z*‐scores. This approach was conceptually similar to a previously described measure of early life SES.^29^

Measures of vascular risk, vascular disease, and early‐life socioeconomic status were harmonized across cohorts using established Rush definitions. Prior work by our group and others supports associations between several of these measures and dementia‐related outcomes, for example, vascular risk burden.^30^, ^31^ Although early‐life SES is associated with late‐life cognition when complemented by neighborhood census data,^32^ such information is not available in all five cohorts.

### 2.4 Statistical analyses

Baseline characteristics were summarized using means (standard deviations [SDs]), medians (interquartile ranges [IQRs]), or counts (percentages), as appropriate. Group differences by sex and race were assessed using chi‐square or Fisher's exact for categorical variables and analysis of variance (ANOVA) or Kruskal–Wallis tests for categorical variables.

We estimated lifetime risk of dementia and MCI from ages 55–105 using non‐parametric cumulative incidence functions that account for competing risks to avoid overestimation. Age was used as the time scale, and delayed entry was accommodated through left truncation at the age at cohort entry.

The target estimand was the cumulative incidence of first dementia (or MCI) diagnosis by a given age, conditional on being alive and free of the outcome at the index age. Death prior to dementia or MCI diagnosis was treated as a competing event. Although subdistribution hazard models such as the Fine–Gray model are commonly used for competing risks, their proportional hazards assumption and limited flexibility under left truncation made them less suitable for the present setting, given the older baseline ages and wide age range of follow‐up. Instead, cumulative incidence functions were estimated nonparametrically using the Aalen–Johansen estimator, which provides model‐free estimates of absolute risk under competing risks and delayed entry.^11^

Pointwise 95% confidence intervals (CIs) were computed using the influence‐function–based variance of the Aalen–Johansen estimator, as implemented in the *prodlim* R package.^33^ This approach avoids proportional hazards assumptions and yields interpretable absolute risk estimates, even in the presence of delayed entry and independent censoring. Although influence‐function–based intervals may be wider in smaller subgroups, they provide conservative and robust inference.

Formally, we defined two competing events $\varepsilon_{i}$: where ε=1 is the diagnosis of interest (dementia or MCI) and ε=2 is the competing event (death without diagnosis). Using age as the time scale, we defined L as the entry age, T as the age at failure, C as the age at censoring, and an indicator δ=I(T<C). We considered an index age $\tau_{0}$ such that $L\geq \tau_{0}$. The observed data for each individual i(i=1,⋯,n) consisted of $\left(L_{i},X_{i},\delta_{i}\cdot \varepsilon_{i},Z_{i}\right)$, where we observe X=T∧C represents the age at diagnosis, death without diagnosis, or censoring, and Z denotes a matrix of covariates (e.g., sex). Lifetime risk was estimated as the cumulative incidence from the index age $\tau_{0}$ (e.g., 55 years) to an advanced age τ (e.g., 105 years):

$$F_{1}\left(\tau |T\rangle \tau_{0}\right)=p(T\leq \tau ,\varepsilon =1\;|T>\tau_{0})=\int_{\tau_{0}}^{\tau }S\left(t|t>\tau_{0}\right)\alpha_{1}\left(t\right)dt$$

where $\alpha_{1}\left(t\right)$ is the cause‐specific hazard function for event 1, and $S\;\left(t|t>\tau_{0}\right)=\;\exp\left(-\int_{\tau_{0}}^{\tau }A\left(u\right)du\right)$ is the overall survival function, where A(t) is the cumulative all‐cause hazard function. Age 55 was the index age. Person‐time was counted until dementia or MCI diagnosis, death, age 105, or administrative censoring (December 2025).

In secondary analyses, lifetime risk was estimated using index ages at 65, 75, and 85 years. Among individuals with incident dementia or MCI, we calculated the median age at diagnosis and the proportion diagnosed between ages 55–74, 75–84, 85–95, and 95–105 years. All analyses were conducted separately for dementia and MCI and stratified by sex and race.

Sex and race differences were formally evaluated using prespecified age‐specific contrasts of cumulative incidence. At ages 75, 85, and 95 years, absolute risk differences were computed as the difference in Aalen–Johansen cumulative incidence between groups (e.g., female minus male, Black minus White), with statistical inference based on Wald‐type 95% CIs and a null hypothesis of zero risk difference. Because analyses used age as the time scale with delayed entry, inference focused on these age‐specific contrasts rather than global curve‐based tests or regression on pseudo‐observations.

Several sensitivity analyses were performed to assess the robustness of lifetime risk estimates.

First, to evaluate the impact of alternative MCI definitions, we compared the primary definition (requiring confirmation at the next available evaluation or death) with: (1) defining incident MCI at the first MCI diagnosis without confirmation, and (2) requiring confirmation within 1‐ and 2‐year windows. Lifetime risk estimates and age‐at‐onset distributions were compared across definitions.

Second, to address potential bias related to incomplete outcome ascertainment and administrative follow‐up, two exclusions were applied in the primary analysis.

Decedents whose last adjudicated clinical evaluation occurred more than 3 years prior to death were excluded to reduce potential outcome misclassification near death.

Participants who had not yet completed a second annual clinical evaluation by the administrative censoring date were excluded because follow‐up was incomplete due to calendar time and would otherwise contribute negligible person‐time while inflating early‐age risk sets.

In separate sensitivity analyses, each group was retained and treated as administratively censored at their last clinical contact. To satisfy left‐truncation requirements in age‐as‐time analyses, a negligible positive follow‐up interval (ε) was added when entry and exit ages coincided.

Third, because analyses using age as time with left truncation assume independence between age at study entry and subsequent event processes conditional on measured covariates, we performed a sensitivity analysis restricting the sample to participants entering between ages 65 and 80 years.

Fourth, to assess whether apparent plateaus in cumulative incidence at advanced ages reflected changes in age‐specific risk versus competing mortality and selective survival, we estimated age‐specific cause‐specific hazards for dementia, MCI, and death without the event. Flexible generalized additive models with penalized splines were fitted using age as the time scale with left truncation. These analyses were descriptive and intended to aid interpretation of cumulative incidence patterns rather than to support formal inference.

Finally, to evaluate the influence of cohort demographic composition, we standardized cumulative incidence curves to the U.S. population age–sex–race distribution using 2019–2023 American Community Survey 5‐year estimates. Participants were stratified by entry age group (55–64, 65–74, 75–84, 85–94, and ≥95 years), sex, and race (Black, White). Within each stratum, cumulative incidence functions accounting for the competing risk of death were estimated using the same methods as in the primary analysis and combined using population weights. Standardized and observed estimates were compared.

In exploratory analyses, we evaluated whether baseline socioeconomic and health‐related factors were associated with incident dementia or MCI using Cox proportional hazards models adjusted for age at baseline, sex, education, and racial/ethnic diversity group. These models estimated cause‐specific hazards, reflecting associations with disease onset among individuals who were alive and event‐free, and were used for etiologic screening rather than estimation of cumulative incidence under competing mortality. For variables showing significant associations, we further estimated the lifetime risk of dementia or MCI stratified by these subgroups. We also explored lifetime risk stratified by Latino versus non‐Latino ethnicity, given the increasing diversity in our cohorts.

All analyses were conducted using R (version 4.4.1), with statistical significance determined at *α* = 0.05.

## 3 RESULTS

### 3.1 Baseline characteristics and incidence of dementia and MCI

Baseline characteristics of participants in the dementia (*n* = 4677) and MCI (*n* = 3990) lifetime risk sets are in Table 1. Across both groups, ≈75% were female, ≈28% Black, and ≈7% Latino among White participants and ≈2% among Black participants. Age distributions (55–64, 65–74, 75–84, 85+) were 212, 1838, 1909, and 718 for dementia; and 196, 1690, 1585, and 519 for MCI. Subgroup comparisons are shown in Table 1 and Tables S1–S2. Baseline age was similar by sex but ≈5 years younger in Black adults.

**TABLE 1:** Characteristics of study participants in estimating lifetime risk of dementia and MCI.

Characteristic	*N* = 4677	*N* = 3990
Age at baseline, years	76.56 (7.77)	75.86 (7.57)
Age at last visit, years	85.49 (7.93)	85.23 (7.98)
Follow‐up, years	8.65 (5.90)	9.08 (5.96)
Female, *n* (%)	3491 (74.6)	3033 (76.0)
White, *n* (%)	3167 (67.7)	2684 (67.3)
Latino, *n* (%)	397 (8.5)	352 (8.8)
Education, years	15.78 (3.95)	15.84 (3.98)
*APOE* ε4 carriers, *n* (%)	968 (26.6)^a^	783 (25.5)^b^
Study, *n* (%)		
ROS	1316 (28.1)	1142 (28.6)
MAP	1977 (42.3)	1621 (40.6)
MARS	773 (16.5)	674 (16.9)
AA	365 (7.8)	332 (8.3)
LATC	246 (5.3)	221 (5.5)
Alive free of dementia/MCI, *n* (%)	1843 (39.4)	1558 (39.0)
Incident cases of dementia/MCI, *n* (%)	1339 (28.6)	1570 (39.3)
Deaths without dementia/MCI, *n* (%)	1495 (32.0)	862 (21.6)
Age at death	88.68 (7.21)	88.60 (7.30)
Interval between age at last and at death (median [IQR])	0.67 [0.35, 0.97]	0.65 [0.34, 0.97]

*Note*: Column 2 corresponds to the analytic dataset used to estimate the lifetime risk of dementia, and Column 3 corresponds to the dataset used to estimate the lifetime risk of MCI. Values are mean (SD) unless otherwise indicated. Race and ethnicity were self‐reported. Race categories reflect White or Black race and include participants of any ethnicity; participants from other racial groups were excluded from race‐stratified analyses due to small sample sizes. Latino ethnicity (yes/no) was recorded separately and examined in exploratory analyses.

Abbreviations: AACORE, African American Clinical Core; *APOE*, apolipoprotein E; IQR: interquartile range; LATC, Latino CORE; MAP, Rush Memory and Aging Project; MARS, Minority Aging Research Study; MCI, mild cognitive impairment; ROS, Religious Orders Study.

^a^ Data missing for 1035 participants.

^b^Data missing for 914 participants.

In the dementia set, over a mean follow‐up of 9 years (range: 1–30 years), there were 1339 incident dementia cases, 1495 deaths without dementia, and 1843 individuals alive and dementia‐free at last visit. The total numbers of participants 55–74, 75–84, 85–94, and 95–105 years of age were 534, 1665, 2053, and 425. The distributions of the three outcomes: censor (alive and dementia‐free), event (incident dementia), and competing risk (death without dementia) across different age groups are presented in Figure S3A, and stratified by sex and race in Figure S3B,C. Dementia incidence was higher in White than in Black participants (32.6% vs 21.6%; *p* < 0.001), and similar across sex (28.7% vs 28.5%). Conversely, death without dementia was more common in White individuals (36.8% vs 23.5%; *p* < 0.001) and male participants (40.6% vs 29.0%; *p* < 0.001). Black participants died ≈6 years earlier than White participants (84 vs 90).

In the MCI set, 1570 participants developed MCI, 862 died without MCI, and 1570 remained MCI‐free. The total numbers of participants 55–74, 75–84, 85–94, and 95–105 years of age were 567, 1561, 1608, and 254, respectively. The distributions of the three outcomes (alive and MCI‐free, incident MCI, and death without MCI) across these age groups are presented in Figure S4A. Stratified distributions by sex and race are shown in Figure S4B,C, respectively. Incidence was higher among White participants (44.3% vs 30.3%; *p* < 0.001), and similar by sex (41.8% in male participants vs 38.6% in female participants). Death without MCI was more common in White participants (24.7% vs 16.7%; *p* < 0.001) and male participants (27.4% vs 19.8%; *p* < 0.001). As with dementia, Black adults died ≈6 years earlier (84 vs 90).

### 3.2 Overall results

#### 3.2.1 Lifetime risk of dementia

**FIGURE 1:**
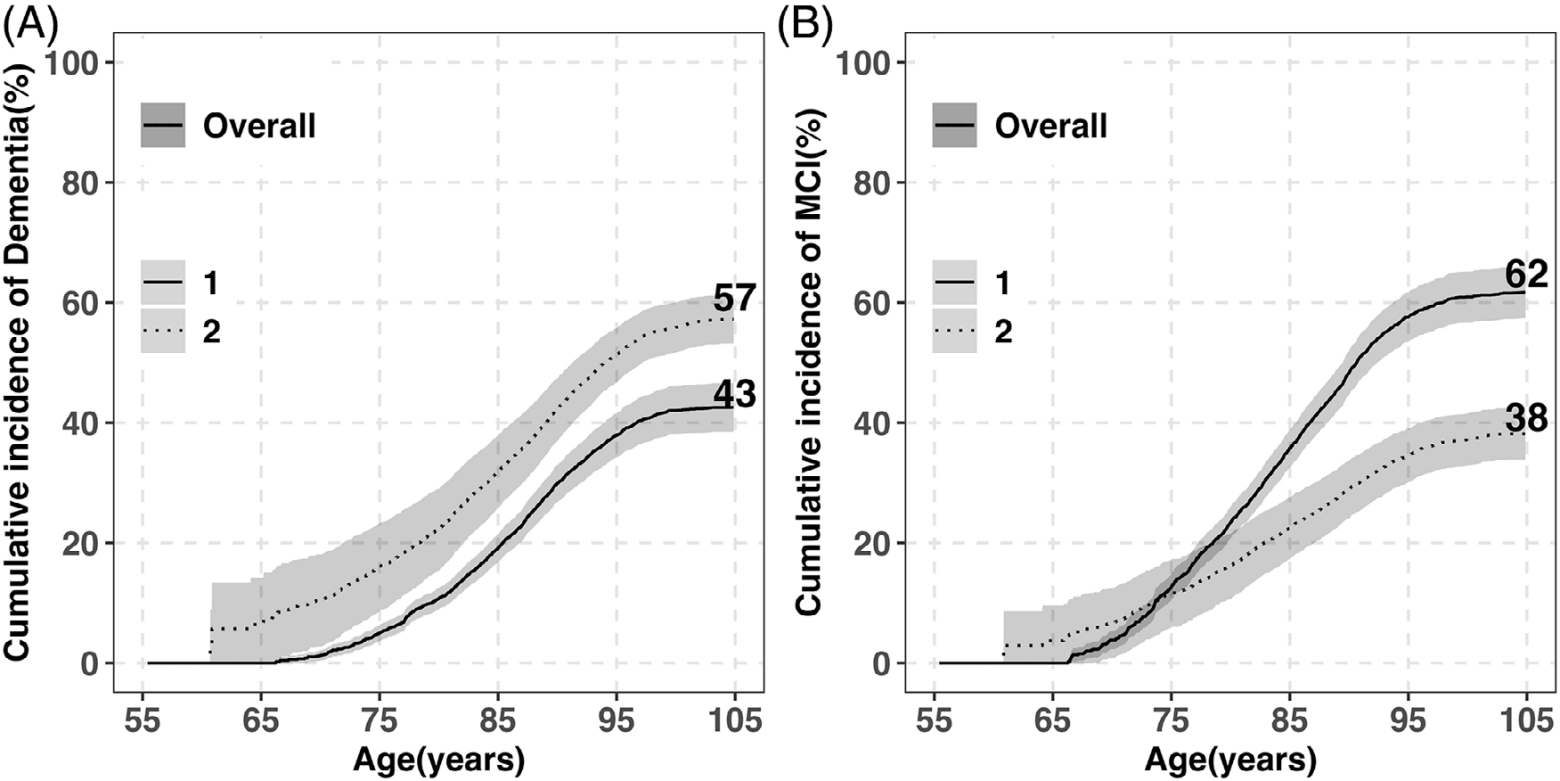
Overall lifetime risk of dementia, MCI, and death without diagnosis (ages 55–105) in the five Rush Alzheimer’s Disease Center cohorts. (A) Cumulative incidence of dementia (solid line) and death without dementia (dashed line) (*n* = 4677). (B) Cumulative incidence of MCI (solid line) and death without MCI (dashed line) (*n* = 3990). Estimates are percentages accounting for competing risk of death. Shaded areas show 95% CIs. Age‐specific cumulative risks overall are provided in Table S3 (dementia/MCI) and Table S4 (death without diagnosis). CI, confidence interval; MCI, mild cognitive impairment.

From age 55 to 105, the lifetime risk of incident dementia was 43% (95% CI: 39–47) (Figure 1A; Table 2). The risk remained low until age 75 (5%), then increased sharply. After age 95, the cumulative incidence curve rises more slowly, increasing from 38% to 43% over the subsequent decade. The cumulative incidence of dementia at each year of age is shown in Table S3. Median age at diagnosis was 88 (IQR: 83–92) (Table 3). Notably, 84% of cases were diagnosed between ages 75 and 95, with 57% occurring between ages 85 and 95.

**TABLE 2:** Lifetime risk of dementia and MCI after select index ages (ages 55, 65, 75, and 85) to 105 years, overall and by sex and race using primary analysis.

	Index age			
	55 years	65 years	75 years	85 years
Lifetime risk of dementia	(*n* = 4677)	(*n* = 4465)	(*n* = 2627)	(*n* = 718)
Overall	43 (39‐47)	46 (43‐48)	49 (46‐51)	45 (41‐50)
Sex				
Female	45 (40‐49)	49 (47‐51)	50 (47‐53)	46 (41‐51)
Male	39 (35‐43)	38 (34‐43)	48 (42‐54)	43 (34‐52)
Race				
White	43 (37‐49)	45 (43‐48)	49 (46‐52)	47 (42‐51)
Black	45 (40‐50)	47 (43‐52)	49 (43‐55)	34 (20‐48)
Lifetime risk of MCI	(*n* = 3990)	(*n* = 3794)	(*n* = 2104)	(*n* = 519)
Overall	62 (57‐66)	64 (62‐66)	68 (66‐71)	64 (58‐69)
Sex				
Female	63 (58‐68)	67 (64, 69)	69 (66‐72)	64 (58‐70)
Male	59 (55‐64)	57 (52‐62)	68 (66‐71)	62 (51‐73)
Race				
White	64 (61‐67)	64 (61‐67)	67 (64‐70)	65 (60‐70)
Black	59 (53‐64)	61 (56‐66)	68 (62‐74)	53 (35‐ 71)

*Note*: Estimates are presented as percentages, representing cumulative incidence through the age of last observation (up to age 105), accounting for the competing risk of death. For subgroups with limited follow‐up at the oldest ages, estimates are reported through the maximum age supported by the data (Black participants through age 102; male participants in MCI analyses through age 104). Corresponding 95% confidence intervals are shown in parentheses.

Abbreviation: MCI, Mild Cognitive Impairment.

**TABLE 3:** Median age at dementia and MCI diagnosis and distribution of diagnosis age, overall and by sex and race.

	Median age [IQR] at diagnosis, years	Percentage diagnosed between ages 55 and 74	Percentage diagnosed between ages 75 and 84	Percentage diagnosed between ages 85 and 95	Percentage diagnosed between ages 95 and 105
Dementia					
Overall	87.59 [82.78, 91.90]	3.9%	26.8%	56.8%	12.5%
Sex					
Female	87.98 [83.13, 92.38]	5.2%	27.9%	54.3%	12.5%
Male	86.36 [82.28, 90.75]	4.4%	37.0%	49.1%	9.2%
Race					
White	88.52 [84.00, 92.70]	2.9%	26.5%	56.6%	13.9%
Black	84.49 [80.02, 88.65]	9.9%	43.1%	42.8%	4.2%
MCI					
Overall	85.62 [80.16, 90.13]	8.4%	34.6%	49.8%	7.3%
Sex					
Female	85.88 [80.51, 90.43]	9.7%	35.3%	47.4%	7.5%
Male	84.53 [79.78, 89.60]	9.2%	44.0%	42.2%	4.5%
Race					
White	86.89 [82.24, 90.91]	5.3%	34.1%	52.0%	8.6%
Black	80.73 [75.96, 85.78]	21.1%	49.0%	28.7%	1.2%

*Note*: Percentages may not total 100% due to rounding.

Abbreviations: IQR, Interquartile range; MCI, Mild Cognitive Impairment

Lifetime risk was related to index ages (Table 2). Among participants who are alive and dementia‐free at age 75 (*n* = 2627), the estimated lifetime risk through age 105 was 49% (95% CI: 46–51), corresponding to ≈930 incident dementia cases observed over follow‐up. Among those alive and dementia‐free at age 85 (*n* = 718), the lifetime risk was 45% (95% CI: 41–50), based on ≈278 incident cases. The modest decline at age 85 likely reflects the smaller number of participants at risk and shorter remaining life expectancy, as indicated by wider CIs.

Between ages 55 and 105, the overall cumulative incidence of death without dementia was 57% (95% CI: 53–61), as shown in Figure 1A. In parallel with the incidence of dementia, the risk of death also plateaued at 51% at age 95, increasing to only 57% over the next decade. The cumulative risks of death at each year of age are provided in Table S4.

#### 3.2.2 Lifetime risk of MCI

Lifetime MCI risk from age 55 to 105 was 62% (95% CI: 57–66), ≈20% higher than dementia risk (Figure 1B; Table 2). The cumulative incidence of MCI increased rapidly after age 65, roughly 10 years earlier than for dementia. In parallel with dementia, the curve rises more slowly after age 95, increasing from 58% to 62% over the next decade. Corresponding cumulative incidence estimates across ages are provided in Table S3. Median age at diagnosis was 86 (IQR: 80–90), about 2 years younger than the median age at dementia diagnosis (Table 3). Eighty‐four percent of MCI cases were diagnosed between ages 75 and 95, with 50% occurring between ages 85 and 95.

MCI risk increased from 62% among participants alive and MCI‐free at age 55 to 68% among those alive and MCI‐free at age 75 (*n* = 2104), corresponding to ≈1033 incident MCI cases over follow‐up (Table 2). Among participants alive and MCI‐free at age 85 (*n* = 519), lifetime MCI risk was 64% (95% CI: 58–69), based on ≈266 incident cases, again reflecting smaller risk sets and shorter life expectancy, as indicated by wider CIs.

The overall cumulative incidence of death without MCI between ages 55 and 95 was 35%, increasing to only 38% (95% CI: 34–42) at age 105 (Figure 1B). Age‐specific cumulative incidence estimates corresponding to this figure are detailed in Table S4.

### 3.3 Sensitivity analyses

Lifetime MCI risk estimates and median age at onset under each definition are reported in Table S5, with cumulative incidence curves shown in Figure S5. Defining MCI at the first diagnosis without confirmation yielded higher lifetime risk estimates and earlier apparent onset, whereas stricter confirmation windows (1‐ and 2‐year) yielded lower risk estimates. The primary definition produced intermediate estimates.

Participants who were excluded due to long gaps between last clinical evaluation and death or not yet reaching the window for an annual follow‐up by the December 2025 data cutoff were older at baseline; sex distributions were similar, whereas racial composition differed modestly (Table S6). Lifetime risk estimates for both dementia and MCI were nearly identical when these participants were retained and administratively censored (Figure S6).

Cumulative incidence curves for participants entering between ages 65 and 80 closely overlapped those from the full analytic sample (Figure S7), indicating robustness of lifetime risk estimates to variation in age at cohort entry.

American Community Survey (ACS)‐standardized cumulative incidence curves closely overlapped observed cohort estimates for both dementia and MCI across most of the age range (Figure S8), indicating that cohort demographic composition did not materially influence lifetime risk estimates.

In spline‐based analyses of age‐specific cause‐specific hazards (Figure S9), hazards for dementia, MCI, and competing mortality continued to increase at advanced ages, suggesting that the slower rise in cumulative incidence after age 95 reflects diminishing risk sets and competing mortality rather than a true plateau in underlying disease risk.

### 3.4 Sex differences in lifetime risk

#### 3.4.1 Lifetime risk of dementia by sex

**FIGURE 2:**
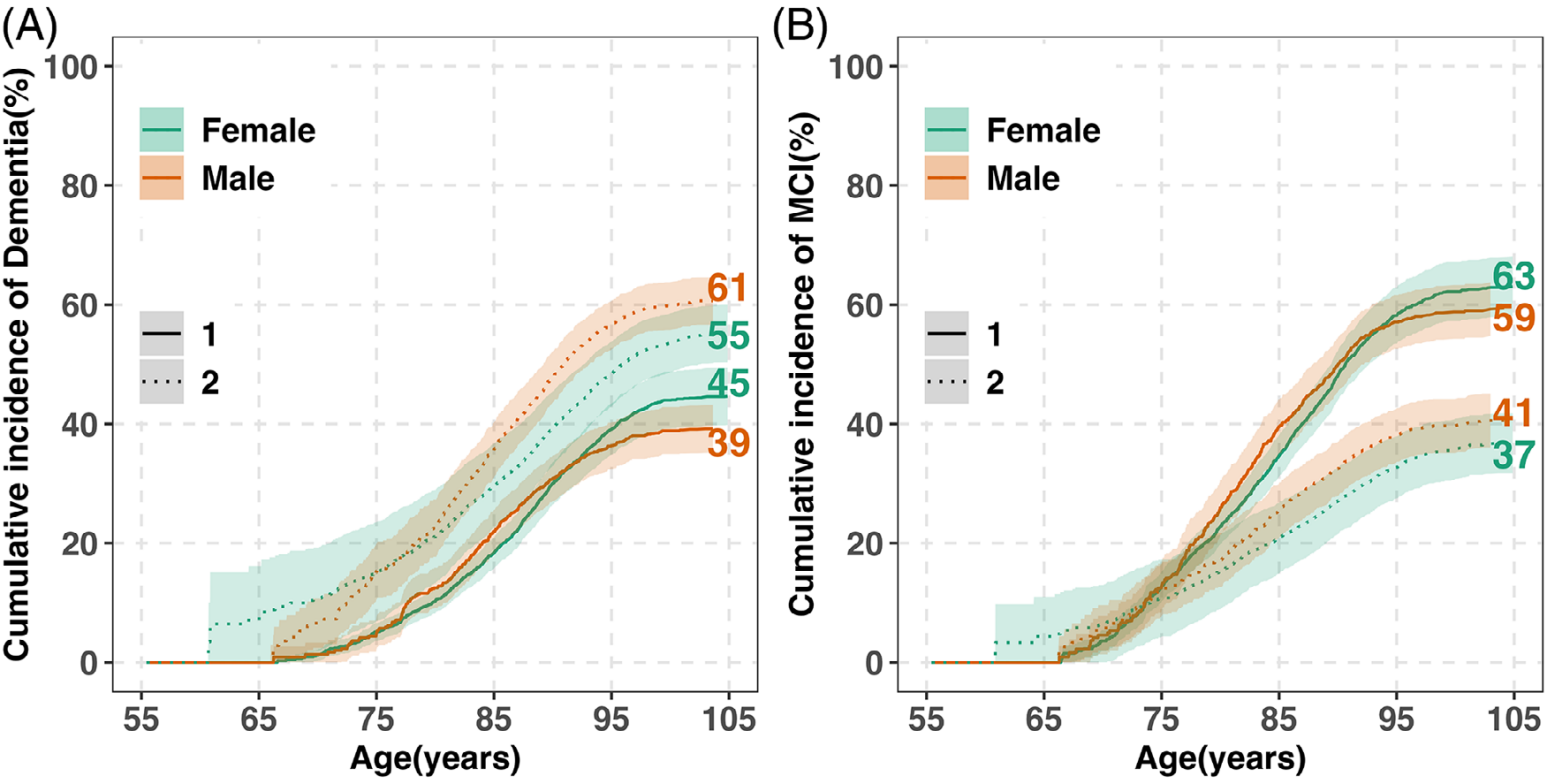
Lifetime risk of dementia, MCI, and death without diagnosis by sex (ages 55–105) in the five Rush cohorts. (A) The cumulative incidence of dementia (solid lines) and death without dementia (dashed lines) is shown for females and males (colored separately). (B) The cumulative incidence of MCI (solid lines) and death without MCI (dashed lines) is also shown for females and males. Estimates are percentages accounting for the competing risk of death. Shaded areas show 95% CIs. Age‐specific cumulative risks by sex are provided in Table S3 (dementia/MCI) and Table S4 (death without diagnosis). CI, confidence interval; MCI, mild cognitive impairment.

From age 55, female participants had a higher lifetime risk of dementia than male participants (45% vs 39%) (Figure 2A; Table S3). Sex differences were most pronounced at advanced ages. The median age at dementia diagnosis was ≈2 years later in female than in male participants (88 vs 86, *p* < 0.01; Table 3). Although most dementia diagnoses occurred after age 75 in both sexes, a greater proportion of female participants were diagnosed between ages 85 and 105 (67% vs 58%), whereas male participants had a higher proportion of diagnoses between ages 75 and 84 (37% vs 28%).

Lifetime risk varied by index age (Table 2). Among participants alive and dementia‐free at age 75, the estimated lifetime risk was 50% in female participants (695/1940) and 48% in male participants (209/542). Among those alive and dementia‐free at age 85, lifetime risk declined to 46% in female participants (235/687) and 43% in male participants (69/176), likely reflecting smaller risk sets and shorter life expectancy, as indicated by wider CIs.

Formal comparisons based on absolute risk differences are summarized in Table S7. The female–male differences were 1.6% at age 75, –2.4% at age 85, and 3.2% at age 95, with CIs crossing zero at all ages.

The cumulative incidence of death without dementia from age 55 to 105 was higher in male than in female participants (61% vs 55%), with differences emerging around age 80 (Figure 2A; Table S4).

#### 3.4.2 Lifetime risk of MCI by sex

The lifetime risk of MCI was higher in female than male participants (63% vs 59%) when estimated from age 55 (Figure 2B; Table S3), with most sex differences emerging after age 90. Median age at MCI diagnosis was ≈2 years later in female than in male participants (86 vs 84 years, *p* < 0.05; Table 3). Most MCI diagnoses occurred after age 75 in both sexes, with a higher proportion between ages 85 and 105 among female participants (55% vs 45%), and a higher proportion between ages 75 and 84 among male participants (44% vs 35%).

Lifetime MCI risk increased with older index ages (Table 2). Among participants alive and MCI‐free at age 75, the lifetime risk was 69% in female participants (786/1610) and 68% in male participants (247/494). Among those alive and MCI‐free at age 85, estimates declined to 64% in female participants (212/414) and 62% in male participants (54/105), consistent with a smaller sample size and reduced remaining lifespan.

The absolute risk differences (female minus male) were 0% at age 75, –4.9% at age 85, and 1.1% at age 95, with CIs crossing zero at all ages (Table S7).

The cumulative incidence of death without MCI from age 55 to 105 was higher in male than in female participants (41% vs 37%) (Figure 2B and Table S4).

### 3.5 Racial differences in lifetime risk

#### 3.5.1 Lifetime risk of dementia by race

**FIGURE 3:**
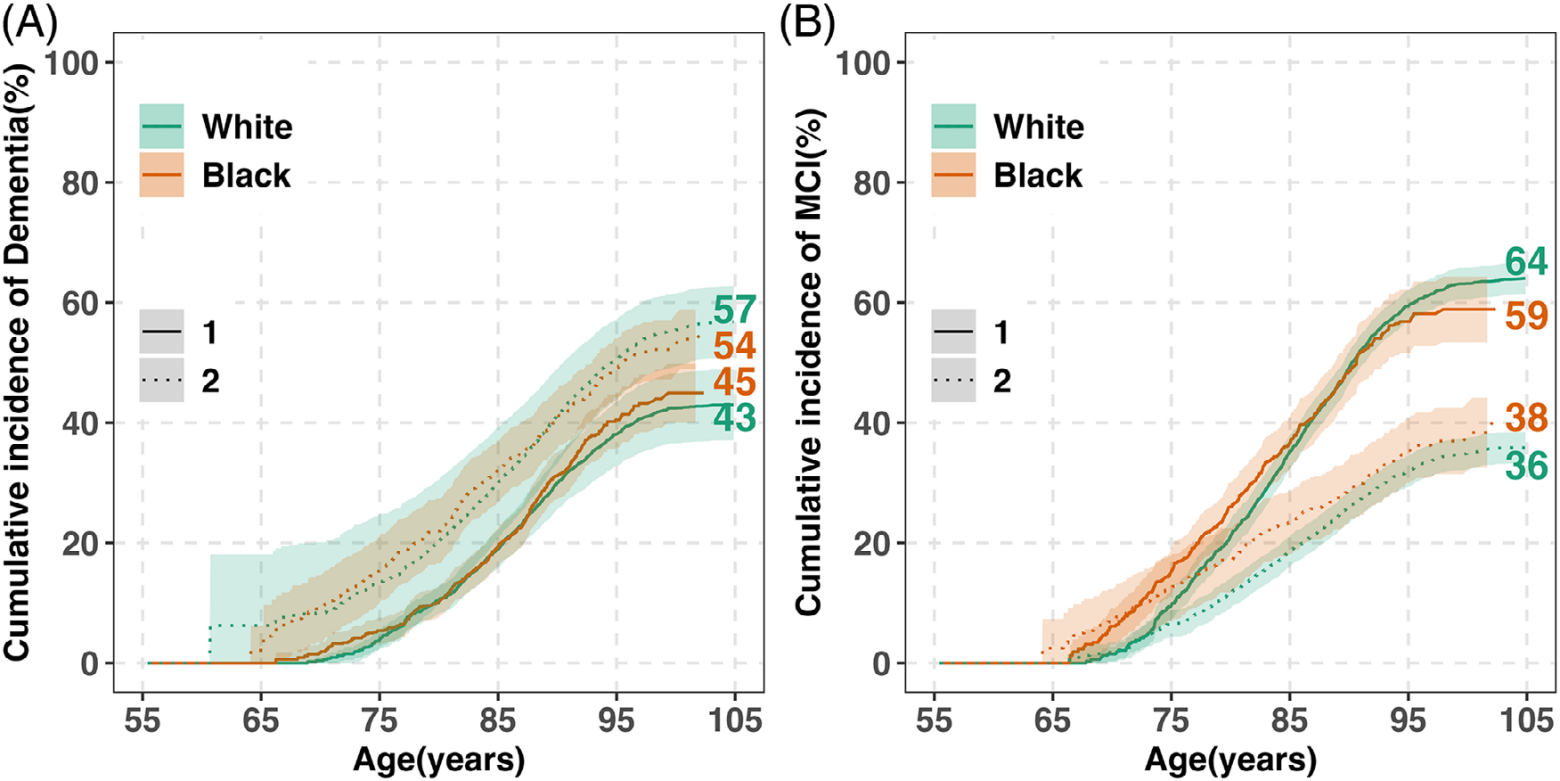
Lifetime risk of dementia and MCI and death without diagnosis by race (ages 55–105) in the five Rush cohorts. (A) The cumulative incidence of dementia (solid lines) and death without dementia (dashed lines) is shown for white and black (colored separately). (B) The cumulative incidence of MCI (solid lines) and death without MCI (dashed lines) is also shown for White and Black. Estimates are percentages accounting for the competing risk of death. Shaded areas show 95% CIs. Age‐specific cumulative risks by race are provided in Table S3 (dementia/MCI) and Table S4 (death without diagnosis). CI, confidence interval; MCI, mild cognitive impairment.

Starting at age 55, the lifetime risk of dementia through age 105 was slightly higher in Black than in White participants (45% vs 43%; Figure 3A; Table S3). However, CIs for these estimates overlapped, and the difference should be interpreted with caution, given reduced precision in smaller subgroups. In addition, projections for Black adults extended only to age 100 due to their younger baseline ages and ages at death. Median age at dementia diagnosis was ≈5 years younger in Black than in White participants (84 vs 89 years, *p* < 0.001; Table 3). A greater proportion of dementia cases in Black adults occurred before age 75 (10% vs 3%) and between ages 75 and 84 (43% vs 27%), compared to White participants.

Lifetime risk differed by index age (Table 2). Among participants alive and dementia‐free at age 75, lifetime risk was 49% in Black participants (132/445) and 49% in White participants (788/2118). Among those alive and dementia‐free at age 85, lifetime risk declined to 34% in Black participants (17/69) and 47% in White participants (260/643), likely reflecting smaller sample sizes and shorter life expectancy in Black participants.

Absolute risk differences by race are shown in Table S7. Black–White differences were small and not statistically significant at ages 75 and 85.

Black individuals had a higher cumulative incidence of death without dementia before age 85 (from ages 55 to 85, 32% vs 30%; Figure 3A; Table S4), with the differences starting at about age 70. However, at older ages, racial differences diminished with a modest cross‐over at about age 90 driven by female participants, a phenomenon reported previously.^34^

#### 3.5.2 Lifetime risk of MCI by race

Starting at age 55, Black adults had a higher lifetime risk of MCI beginning after age 65, but a lower risk after age 90 compared to White adults. From ages 55 to 75, the risk was 15% in Black participants versus 10% in White participants, and from ages 55 to 105, 59% versus 64% (Figure 3B; Table S3). However, CIs overlapped across much of the age range, and subgroup comparisons, especially among the oldest‐old, should be interpreted with caution. Projections for Black participants extended only to age 100 due to younger baseline ages and shorter life expectancy, and the wider confidence intervals at older ages reflect reduced precision in this subgroup. Median age at MCI diagnosis was ≈6 years younger in Black than White participants (81 vs 87 years, *p* < 0.001; Table 3). A greater proportion of MCI diagnoses in Black participants occurred before age 75 (21% vs 5%) and between ages 75 and 84 (49% vs 34%).

Lifetime MCI risk varied by index age (Table 2). Among participants alive and MCI‐free at age 75, lifetime risk was 68% in Black participants (151/351) and 67% in White participants (863/1697). Among those alive and MCI‐free at age 85, estimates declined to 53% in Black participants (19/51) and 65% in White participants (245/463), reflecting reduced precision due to smaller risk sets.

Risk differences (Black minus White) were statistically significant at age 75 (5.7% higher in Black participants) but not statistically significant at age 85 (Table S7).

Death without MCI was more frequent in Black adults, with a cumulative incidence through age 105 of 38% compared to 36% in White adults (Figure 3B and Table S4); differences became apparent beginning around age 65.

### 3.6 Exploratory lifetime risk analyses by baseline morbidity, socioeconomic status, and Latino ethnicity

#### 3.6.1 Morbidity and socioeconomic factors

In exploratory analyses, we examined whether baseline morbidity and socioeconomic characteristics were associated with dementia and MCI incidence using Cox models adjusted for age at baseline, sex, diversity group, and education. Education, most baseline morbidity and socioeconomic indicators were not significantly associated with dementia or MCI incidence. Only stroke history was significantly associated with increased risk of dementia and MCI. (See the results in Table S8.) We then estimated lifetime risk stratified by stroke history. Participants with stroke history, for example, had earlier onset and greater cumulative incidence of both dementia and MCI compared to those without. Detailed risk estimates by subgroup are provided in Tables S9 and S10, and in Figure S10.

#### 3.6.2 Latino ethnicity

We also estimated the cumulative incidence of dementia and MCI by Latino ethnicity. Latino participants were younger at baseline and less likely to have a clinical diagnosis over the follow‐up period. However, lifetime risk projections were higher compared to non‐Latino participants and more uncertain due to a smaller sample size and fewer events. Nonetheless, stratified estimates for Latino versus non‐Latino groups are provided in Tables S9 and S10, and in Figure S11 to improve representation and guide future analyses as the cohort grows.

## 4 DISCUSSION

This study provides estimates of the lifetime risk of dementia and MCI from age 55 to 105, using high‐quality data from five harmonized longitudinal cohorts. By incorporating competing risk and left truncation methods, our findings build upon previous estimates. In the absence of effective public health strategies, our findings suggest that nearly half of older adults will develop dementia, whereas close to two thirds will develop MCI during their lifetime, with notable differences across sex and race. Actual numbers may be lower, as some evidence suggests a slight decline in age‐specific dementia prevalence over the past four decades,^35^ likely reflecting improvements in education, cardiovascular health, and lifestyle. However, rising life expectancy and persistent racial differences in health care access may offset these gains.^36^ Although the figures suggest that dementia incidence may plateau in the oldest old, this pattern should be interpreted cautiously and does not, by itself, imply a biological decline in risk. Rising cause‐specific hazards and a flattening cumulative incidence can coexist because cumulative incidence reflects lifetime risk under competing mortality. Prior studies reported that dementia incidence increases with age but may peak and decline among the extremely old,^37^ or rise at a slower rate beyond age 85.^38^ Our earlier work showed that AD pathology peaked at around age 95 and leveled off thereafter,^39^ although findings across cohorts remain inconsistent.^40^, ^41^, ^42^

### 4.1 Lifetime risk compared with previous studies and clinical implications

Our most striking finding is the high lifetime burden of MCI, which begins to rise as early as age 65, nearly a decade before dementia onset. This extended pre‐dementia phase represents a critical window for early detection and intervention. Of note, lifetime risk estimates should be interpreted as residual risk conditional on being alive and free of the outcome at a given age, rather than as a deterministic prognosis for individuals. For example, among participants alive and dementia‐free at age 75, approximately half developed dementia by age 105, underscoring the substantial residual risk even at older ages. From a public health perspective, the magnitude of MCI risk, affecting over half of the population by age 105, suggests a growing need for resource allocation toward early detection infrastructure and community‐based cognitive health programs. This is the first study, to our knowledge, to estimate cumulative MCI risk from age 55 to 105 while accounting for competing mortality and left truncation, offering novel insights into the timing and scale of MCI burden in aging populations. Prior estimates from the Health and Retirement Study (HRS) reported higher lifetime MCI risk,^13^ likely reflecting broader diagnostic criteria and younger sample ages. By requiring diagnostic confirmation, our primary definition likely reduced misclassification from transient impairment, yielding more conservative and clinically meaningful estimates.

These results have several clinical and translational implications. Individuals with persistent MCI may represent ideal targets for preventive trials and interventions. Groups at elevated risk of early‐onset or prolonged MCI, such as women, individuals with stroke history, and Black adults, may benefit from more intensive monitoring. The extended pre‐dementia phase observed here highlights the importance of timely detection to support care planning and lifestyle interventions. Recent guidelines encourage brief cognitive assessments in primary care, especially for underserved and high‐risk populations.^43^ Implementation studies have shown that identifying MCI early improves management of reversible contributors, reduces fall risk and hospitalizations, and facilitates access to support services.^44^ Nonetheless, over 90% of older adults with MCI remain undiagnosed, with lower recognition rates among Black and low‐income individuals,^2^ reflecting a significant gap in care delivery.

In addition to MCI, we observed a high lifetime risk of dementia, especially between ages 75 and 95. The risk increased progressively with older index ages (65 and 75), further supporting the importance of long‐term cognitive follow‐up in aging populations. Compared to prior estimates, our dementia risk was similar or higher,^7^, ^12^, ^13^, ^15^, ^17^, ^45^ which may reflect differences in population age distribution, diagnostic methods, follow‐up frequency, or analytic methods. For instance, our findings build on those from the Atherosclerosis Risk in Communities (ARIC) study^12^ by estimating risk beyond age 95 and reveal slightly higher risk prior to age 84. Compared to the HRS,^13^ our dementia estimates were higher but had a later onset. These contrasts emphasize the importance of consistent longitudinal tracking and harmonized diagnosis in producing comparable estimates across cohorts.

### 4.2 Sex differences in lifetime risk

Sex‐specific analyses revealed no clear differences in the incidence of dementia or MCI, but female participants exhibited a higher overall lifetime risk of both outcomes, largely driven by higher mortality among male participants prior to dementia and MCI onset. This pattern aligns with multiple prior studies, including ARIC (48% vs 35%),^12^ the Framingham Heart Study^7^, ^15^ (23% vs 14%), the HRS (37% vs 24%),^13^ and a biomarker‐based study (27% vs 21% at age 75).^16^ Our lifetime estimates were similar to ARIC and generally higher than others, which may reflect differences in population age structures, follow‐up duration, mortality rates, and diagnostic approaches.

Survival differences alone may not fully explain observed sex disparities. Female participants generally demonstrate higher baseline cognitive performance, consistent with greater reserve, yet several studies suggest more rapid decline once impairment begins.^46^ Biological factors, including greater tau pathology, higher white matter burden, and hormonal changes, may increase vulnerability after onset.^47^, ^48^ Social and structural factors, such as caregiving roles, depression, lower early‐life educational quality, and delayed symptom recognition, may also contribute.^49^, ^50^ In addition, age, apolipoprotein E (*APOE*) ε4, and vascular risk factors appear to exert sex‐specific effects.^51^ Emerging evidence of more aggressive tau pathology in female participants^52^ further supports the need for sex‐stratified approaches in dementia research.

### 4.3 Racial differences in lifetime risk

Racial disparities in lifetime dementia and MCI risk were observed. Black participants had a higher risk of both dementia and MCI before age 85, but a lower risk of MCI after age 95, likely due to selective survival. These patterns may reflect younger baseline ages, higher competing mortality, and the possibility that long‐lived Black participants constitute a particularly resilient subgroup. Although our findings are consistent with the ARIC study,^12^ the magnitude of racial differences was smaller after adjusting for left truncation. Prior studies, particularly those with a sample restricted to survivors to age 90, reported wider disparities but were potentially biased by survival conditioning.^42^

Beyond survival dynamics, racial disparities in cognitive aging may reflect higher rates of vascular/metabolic conditions, reduced health care access, chronic stress, and socioeconomic factors.^53^, ^54^ Our results extend earlier studies^1^, ^13^, ^40^, ^55^ that focused primarily on prevalence or relative risk without considering competing risks. Although barriers to care and biomarker differences likely contribute,^56^ our results emphasize the need for life‐course, context‐sensitive approaches and larger, more diverse cohorts to better characterize racial disparities in lifetime dementia and MCI risk, particularly in the oldest‐old.

### 4.4 Other subgroup differences in lifetime risk

Among exploratory subgroups, stroke history was the strongest predictor of earlier onset and higher cumulative risk of both dementia and MCI, consistent with prior meta‐analytic evidence.^57^ Although Latino participants were underrepresented, stratified estimates suggested higher and more uncertain lifetime risks, underscoring the need for greater inclusion of Hispanic/Latino populations in longitudinal aging studies.

### 4.5 Strengths and limitations

This study has several strengths. We used data from five large, harmonized cohorts of older adults without known dementia at baseline, followed annually for up to 30 years. Annual evaluations allowed detection of rapidly progressive cases that might be missed in studies with longer follow‐up intervals. Follow‐up was excellent, with over 90% retention of survivors and minimal attrition, and consistent clinical diagnostic protocols across cohorts enhances comparability. Our conservative MCI definition likely reduced misclassification, thereby increasing diagnostic specificity. Analyses accounted for delayed entry and competing risks using the nonparametric Aalen–Johansen estimator for cumulative incidence. Although this estimator admits an influence‐function (pseudo‐observation) representation for variance estimation, we did not perform regression modeling of pseudo‐observations; therefore, link functions, working correlation structures, and jackknife regression variance estimators were not applicable. Confidence intervals may widen in smaller subgroups or at the oldest ages, reflecting diminishing risk sets rather than model instability, and subgroup comparisons should be interpreted cautiously when intervals overlap. Despite these strengths, limitations exist. Our sample was primarily Black and White, urban‐dwelling, and relatively well‐educated, with limited Latino and no Asian American participants, which may limit generalizability. However, sensitivity analyses standardizing estimates to the U.S. age–sex–race distribution yielded results very similar to observed estimates, suggesting that findings were not driven by demographic composition. Missing data were not imputed, although high retention and the analytic framework mitigate potential bias. Inverse probability weighting was not applied; however, the nonparametric approach and high follow‐up completeness reduce sensitivity to informative censoring. Measures of vascular and socioeconomic factors relied on baseline and partially self‐reported data, biasing associations toward attenuation. Genetic and cohort effects (e.g., *APOE* genotype, secular trends) were not examined here, although separate analyses are underway. Although exclusions related to incomplete follow‐up could introduce selection bias, sensitivity analyses using alternative censoring assumptions yielded nearly identical lifetime risk curves. Finally, the slowing of cumulative incidence after age 95 should be interpreted with caution. Spline‐based analyses showed continued increases in dementia/MCI and mortality hazards, suggesting that this pattern reflects selective survival and competing mortality rather than declining biological risk. Lifetime risk estimates were robust to restriction by age at entry.

## CONFLICT OF INTEREST STATEMENT

The authors report no relevant disclosures. Any author disclosures are available in the Supporting Information.

## CONSENT STATEMENT

All participants provided written informed consent; individual studies were approved by an institutional review board at Rush University Medical Center.

## Supporting information



Supporting information

Supporting information

## Data Availability

Data used for this study can be requested for research purposes through the Rush Alzheimer's Disease Center Research Resource Sharing Hub (https://www.radc.rush.edu/), subject to institutional approval and data‐use agreements. All analysis code used to generate the results in this study is available at [https://github.com/Lian939/lifetime‐risk‐dementia‐mci]. The repository includes scripts implementing cumulative incidence estimation with left truncation and competing risks using the Aalen–Johansen framework.
