# Supplementary material for: Lifetime risk of incident dementia and incident mild cognitive impairment in older adults
Source: Alzheimers Dement. 2026 Feb 3;22(2):e71173. doi: 10.1002/alz.71173 (PMC12865328; doi:10.1002/alz.71173)
Supplement: Supplementary file 1 — Supporting information [file ALZ-22-e71173-s002.docx]

**Lifetime risk of incident dementia and incident mild cognitive impairment in older adults**

Supplementary Materials

**Table S1 Characteristics of Study Participants by Sex and Race in Estimating Lifetime Risk of Dementia.**

| **Characteristic** | **Sex** | | **Race** | |
| --- | --- | --- | --- | --- |
|  | **Female** | **Male** | **White** | **Black** |
| Age at baseline, years | 76.50 (7.80) | 76.76 (7.67) | 78.36 (7.66)* | 72.98 (6.42)* |
| Age at last visit, years | 85.64 (8.05)* | 85.04 (7.55)* | 87.29 (7.46)* | 82.25 (7.46)* |
| Follow up years | 8.85 (5.87)* | 8.06 (5.93)* | 8.80 (6.11) | 8.69 (5.60) |
| Female, n (%) | -- | -- | 2293 (72.4)* | 1045 (79.8)* |
| White, n (%) | 2293 (49.0)* | 874 (73.7)* | -- | -- |
| Education, years | 15.53 (3.80)* | 16.49 (4.28)* | 16.38 (3.81)* | 15.03 (3.40)* |
| *APOE* ε4 carriers ^a^, n (%) | 739 (27.2) | 229 (24.7) | 574 (22.9)* | 377 (36.5)* |
| Study, n (%) |  | * |  | * |
| ROS | 940 (26.9) | 376 (31.7) | 1213 (38.3) | 89 (6.8) |
| MAP | 1458 (41.8) | 519 (43.8) | 1859 (58.7) | 94 (7.2) |
| MARS | 598 (17.1) | 175 (14.8) | 0 (0.0) | 771 (58.9) |
| AA | 301 (8.6) | 64 (5.4) | 2 (0.1) | 354 (27.0) |
| LATC | 194 (5.6) | 52 (4.4) | 93 (2.9) | 1 (0.1) |
| Alive free of dementia, n (%) | 1477 (42.3)* | 366 (30.9)* | 970 (30.6)* | 718 (54.9)* |
| Incident cases of dementia, n (%) | 1001 (28.7) | 338 (28.5) | 1032 (32.6)* | 283 (21.6)* |
| Deaths without dementia,  n (%) | 1013 (29.0)* | 482 (40.6)* | 1165 (36.8)* | 308 (23.5)* |
| Age at death | 89.19 (7.29)* | 87.49 (6.87)* | 89.79 (6.63)* | 84.21 (7.63)* |
| Interval between age at last and at death, (median [IQR]) | 0.68 [0.36, 0.99] | 0.64 [0.32, 0.96] | 0.64 [0.34, 0.94]* | 0.75 [0.40, 1.19]* |

Values are mean (SD) and for the entire analytic sample unless otherwise stated.

Abbreviations: ROS, Religious Orders Study; MAP, Memory and Aging Project; MARS, Minority Aging Research Study; AACORE, Clinical Core; LATC, Latino Core; *APOE*, Apolipoprotein E; IQR: Interquartile range

^a^ Data missing for 901 participants.

**P*<0.05.

**Table S2 Characteristics of Study Participants by Sex and Race in Estimating Lifetime Risk of MCI.**

| **Characteristic** | **Sex** | | **Race** | |
| --- | --- | --- | --- | --- |
|  | **Female** | **Male** | **White** | **Black** |
| Age at baseline, years | 75.95 (7.62) | 75.58 (7.41) | 77.56 (7.56)* | 72.50 (6.12)* |
| Age at last visit, years | 85.42 (8.05)* | 84.61 (7.71)* | 87.06 (7.57)* | 81.97 (7.33)* |
| Follow up years | 9.17 (5.91) | 8.79 (6.10) | 9.36 (6.20) | 8.87 (5.56) |
| Female, n (%) | -- | -- | 1982 (73.8)* | 914 (81.2)* |
| White, n (%) | 1982 (49.7)* | 702 (73.4)* | -- | -- |
| Education, years | 15.61 (3.81)* | 16.56 (4.38)* | 16.46 (3.83)* | 15.10 (3.36)* |
| *APOE* ε4 carriers ^a^, n (%) | 615 (26.3) | 168 (22.6) | 453 (21.4)* | 315 (36.0)* |
| Study, n (%) |  | * |  | * |
| ROS | 821 (27.1) | 321 (33.5) | 1069 (39.8) | 60 (5.3) |
| MAP | 1230 (40.6) | 391 (40.9) | 1530 (57.0) | 71 (6.3) |
| MARS | 531 (17.5) | 143 (14.9) | 0 (0.0) | 672 (59.7) |
| AA | 278 (9.2) | 54 (5.6) | 2 (0.1) | 322 (28.6) |
| LATC | 173 (5.7) | 48 (5.0) | 83 (3.1) | 1 (0.1) |
| Alive free of MCI, n (%) | 1263 (41.6)* | 295 (30.8)* | 833 (31.0)* | 597 (53.0)* |
| Incident cases of MCI, n (%) | 1170 (38.6) | 400 (41.8) | 1189 (44.3)* | 341 (30.3)* |
| Deaths without MCI, n (%) | 600 (19.8)* | 262 (27.4)* | 662 (24.7)* | 188 (16.7)* |
| Age at death | 89.15 (7.33)* | 87.22 (7.06)* | 89.74 (6.71)* | 83.81 (7.62)* |
| Interval between age at last and at death, (median [IQR]) | 0.66 [0.34, 0.97] | 0.63 [0.34, 0.96] | 0.63 [0.33, 0.94]* | 0.74 [0.40, 1.20]* |

Values are mean (SD) and for the entire analytic sample unless otherwise stated.

Abbreviations: MCI, Mild Cognitive Impairment; ROS, Religious Orders Study; MAP, Memory and Aging Project; MARS, Minority Aging Research Study; AACORE, Clinical Core; LATC, Latino Core; *APOE*, Apolipoprotein E; IQR: Interquartile range

^a^ Data missing for 790 participants.

**P*<0.05.

**Table S3 The cumulative incidence of dementia or MCI at each year of age, overall, by sex and race**

| **Cumulative incidence** | **Overall** | **Sex** | | **Race** | |
| --- | --- | --- | --- | --- | --- |
|  |  | **Female** | **Male** | **White** | **Black** |
| Cumulative incidence of Dementia |  |  |  |  |  |
| Age 75 years | 5 (4, 6) | 5 (4, 7) | 5 (2, 8) | 4 (2, 5) | 5 (3, 8) |
| Age 85 years | 19 (17, 21) | 18 (16, 21) | 22 (18, 26) | 19 (16, 22) | 19 (16, 23) |
| Age 95 years | 38 (34, 42) | 39 (35, 43) | 36 (32, 40) | 38 (33, 43) | 40 (36, 45) |
| Age 105 years | 43 (39, 47) | 45 (40, 49) | 39 (35, 43) | 43 (37, 49) | 45 (40, 50) |
| Cumulative incidence of MCI |  |  |  |  |  |
| Age 75 years | 13 (11, 15) | 13 (10, 15) | 13 (9, 17) | 10 (7, 12) | 15 (12, 19) |
| Age 85 years | 36 (33, 39) | 35 (31, 38) | 40 (35, 44) | 35 (32, 38) | 37 (32, 41) |
| Age 95 years | 58 (54, 62) | 58 (53, 63) | 57 (53, 62) | 59 (57, 62) | 57 (52, 62) |
| Age 105 years | 62 (57, 66) | 63 (58, 68) | 59 (55, 64) | 64 (61, 67) | 59 (53, 64) |

Estimates are reported as percentages and indicate the cumulative incidence at the age of last observation (up to age 105 years) after accounting for the competing risk of death. For Black participants, estimates are reported through age 102 due to limited follow-up at older ages. The 95% CIs are reported in parentheses.

**Table S4 The cumulative incidence of death without dementia or MCI at each year of age, overall, by sex and race**

| **Cumulative incidence** | **Overall** | **Sex** | | **Race** | |
| --- | --- | --- | --- | --- | --- |
|  |  | **Female** | **Male** | **White** | **Black** |
| Cumulative incidence of Death without Dementia |  |  |  |  |  |
| Age 75 years | 16 (9, 23) | 15 (7, 24) | 15 (11, 20) | 14 (2, 25) | 16 (10, 21) |
| Age 85 years | 32 (26, 38) | 30 (22, 37) | 36 (31, 40) | 30 (21, 39) | 32 (27, 37) |
| Age 95 years | 51 (47, 56) | 48 (43, 54) | 56 (52, 61) | 50 (44, 57) | 49 (44, 54) |
| Age 105 years | 57 (53, 61) | 55 (50, 60) | 61 (57, 65) | 57 (51, 63) | 54 (49, 59) |
| Cumulative incidence of death without of MCI |  |  |  |  |  |
| Age 75 years | 12 (6, 17) | 11 (4, 17) | 12 (8, 17) | 7 (4, 9) | 12 (7, 18) |
| Age 85 years | 22 (17, 28) | 21 (15, 27) | 25 (21, 30) | 18 (16, 21) | 23 (18, 29) |
| Age 95 years | 35 (30, 39) | 33 (27, 38) | 38 (34, 43) | 32 (29, 34) | 35 (30, 41) |
| Age 105 years | 38 (34, 42) | 37 (32, 42) | 41 (36, 45) | 36 (33, 38) | 38 (33, 44) |

Estimates are reported as percentages and indicate the cumulative incidence at the age of last observation (up to age 105 years). For Black participants, estimates are reported through age 102 due to limited follow-up at older ages. The 95% CIs are reported in parentheses.

**Table S5. Sensitivity analyses of lifetime MCI risk under alternative case definitions**

| **MCI Definition** | **Lifetime risk to age 105 (%)** | **Median age at onset (IQR)** | **Events (n)** |
| --- | --- | --- | --- |
| Primary  (confirmed at next evaluation) | 61.7 (57.4–66.0) | 85.6 (80.2–90.1) | 1570 |
| First MCI (no confirmation) | 94.2 (87.1–100.0) | 82.1 (76.4–87.4) | 2229 |
| Confirmed within 1 year | 56.6 (52.6–60.7) | 86.7 (81.5–91.1) | 1408 |
| Confirmed within 2 years | 63.8 (59.4–68.2) | 85.4 (80.1–90) | 1605 |

Lifetime risk represents cumulative incidence through age 105, conditional on being alive and MCI-free at baseline. Estimates account for delayed entry and the competing risk of death using the Aalen–Johansen estimator. Median age at onset is reported among participants with incident MCI.

**Table S6. Characteristics of participants excluded due to follow-up limitations**

|  | **Primary analytic sample** | **Excluded: Long-gap decedents (>3y)** | **Excluded: no second annual evaluation (alive)** |
| --- | --- | --- | --- |
| N | 4677 | 280 | 375* |
| Age at baseline | 76.6 (7.8) | 78.9 (6.7) | 77.1 (7.7) |
| Age at death | 88.7 (7.2) | 90.9 (6.5) | — |
| Female | 3491 (74.6%) | 215 (76.8%) | 299 (79.7%) |
| Male | 1186 (25.4%) | 65 (23.2%) | 76 (20.3%) |
| White | 3167 (70.8%) | 226 (83.1%) | 226 (66.3%) |
| Black | 1309 (29.2%) | 46 (16.9%) | 115 (33.7%) |

* Three excluded participants had baseline age <55 years and were excluded to support age-based left truncation.

**Table S7. Formal comparisons based on absolute risk differences**

| **Outcome** | **Comparison** | **Age 75** | **Age 85** | **Age 95** |
| --- | --- | --- | --- | --- |
| Dementia | Female minus Male | 1.6 (-2.0, 5.2) | -2.4 (-7.2, 2.4) | 3.2 (-2.9, 9.3) |
| Dementia | Black minus White | 0.1 (-4.1, 4.2) | -0.3 (-5.6, 5.1) | 2.0 (-5.4, 9.4) |
| MCI | Female minus Male | 0.0 (-4.8, 4.8) | -4.9 (-10.7, 0.9) | 1.1 (-5.4, 7.6) |
| MCI | Black minus White | 5.7 (1.4, 10.0) | 1.4 (-3.7, 6.6) | -2.5 (-8.4, 3.3) |

**Table S8 Cause-Specific Cox Regression for Incident Dementia or MCI**

|  | **Incident Dementia** | | **Incident MCI** | |
| --- | --- | --- | --- | --- |
| **Variable** | Hazard Ratio (95% CI) | p-value | Hazard Ratio (95% CI) | p-value |
| Age at baseline | 1.12 (1.11–1.13) | <0.001 | 1.11 (1.10–1.12) | <0.001 |
| Male | 1.10 (0.97–1.26) | 0.152 | 1.15 (1.01–1.29) | 0.029 |
| Non-Latino Black | 1.06 (0.91–1.23) | 0.456 | 1.04 (0.90–1.20) | 0.574 |
| Latino | 1.40 (1.07–1.84) | 0.015 | 1.44 (1.14–1.83) | 0.002 |
| Education years | 1.00 (0.98–1.01) | 0.619 | 1.01 (0.99–1.02) | 0.258 |
| Stroke history | 1.41 (1.16–1.73) | <0.001 | 1.34 (1.10–1.63) | 0.003 |

Note: Cause-Specific Cox Regression Model Adjusted for age, sex, race, education, and cardiovascular disease

**Table S9 The cumulative incidence of dementia or MCI at each year of age by Latino ethnicity and stroke history**

| **Cumulative incidence** | **Latino ethnicity** | | **Stroke history** | |
| --- | --- | --- | --- | --- |
|  | **Non-Latino** | **Latino** | **No** | **Yes** |
| Cumulative incidence of Dementia |  |  |  |  |
| Age 75 years | 4 (3, 6) | 10 (5, 15) | 5 (3, 6) | 11 (2, 19) |
| Age 85 years | 19 (17, 21) | 26 (17, 34) | 18 (15, 21) | 28 (18, 38) |
| Age 95 years | 39 (37, 41) | 43 (31, 55) | 37 (32, 42) | 39 (27, 50) |
| Age 105 years | 44 (41, 46) | 49 (36, 62) | 42 (36, 47) | 41 (29, 52) |
| Cumulative incidence of MCI |  |  |  |  |
| Age 75 years | 12 (10, 14) | 20 (12, 27) | 12 (9, 14) | 26 (11, 41) |
| Age 85 years | 35 (33, 38) | 47 (37, 57) | 35 (31, 39) | 49 (36, 62) |
| Age 95 years | 58 (55, 61) | 65 (53, 78) | 56 (51, 62) | 62 (51, 73) |
| Age 105 years | 62 (60, 65) | 65 (53, 78) | 60 (55, 66) | 64 (53, 75) |

Estimates are reported as percentages and indicate the cumulative incidence at the age of last observation (up to age 105 years) after accounting for the competing risk of death. For Latino participants, estimates are reported through age 97 due to limited follow-up at older ages; for participants with stroke history, estimates are reported through age 102 due to limited follow-up at older ages. The 95% CIs are reported in parentheses.

**Table S10 The cumulative incidence of death without dementia or MCI at each year of age by Latino ethnicity and stroke history**

| **Cumulative incidence** | **Latino ethnicity** | | **Stroke history** | |
| --- | --- | --- | --- | --- |
|  | **Non-Latino** | **Latino** | **No** | **Yes** |
| Cumulative incidence of Death without Dementia |  |  |  |  |
| Age 75 years | 12 (9, 15) | 20 (3, 38) | 18 (8, 27) | 26 (10, 43) |
| Age 85 years | 29 (26, 32) | 32 (16, 47) | 33 (25, 41) | 45 (31, 59) |
| Age 95 years | 49 (47, 52) | 49 (36, 62) | 52 (46, 58) | 57 (45, 69) |
| Age 105 years | 56 (54, 58) | 50 (37, 63) | 58 (53, 63) | 59 (48, 71) |
| Cumulative incidence of death without of MCI |  |  |  |  |
| Age 75 years | 10 (7, 13) | 14 (0, 27) | 13 (5, 21) | 16 (5, 26) |
| Age 85 years | 21 (18, 24) | 19 (6, 32) | 24 (17, 31) | 27 (16, 38) |
| Age 95 years | 34 (31, 37) | 32 (20, 44) | 36 (30, 42) | 35 (24, 46) |
| Age 105 years | 38 (35, 40) | 33 (21, 46) | 40 (34, 45) | 36 (25, 47) |

Estimates are reported as percentages and indicate the cumulative incidence at the age of last observation (up to age 105 years). For Latino participants, estimates are reported through age 97 due to limited follow-up at older ages; for participants with stroke history, estimates are reported through age 102 due to limited follow-up at older ages. The 95% CIs are reported in parentheses.


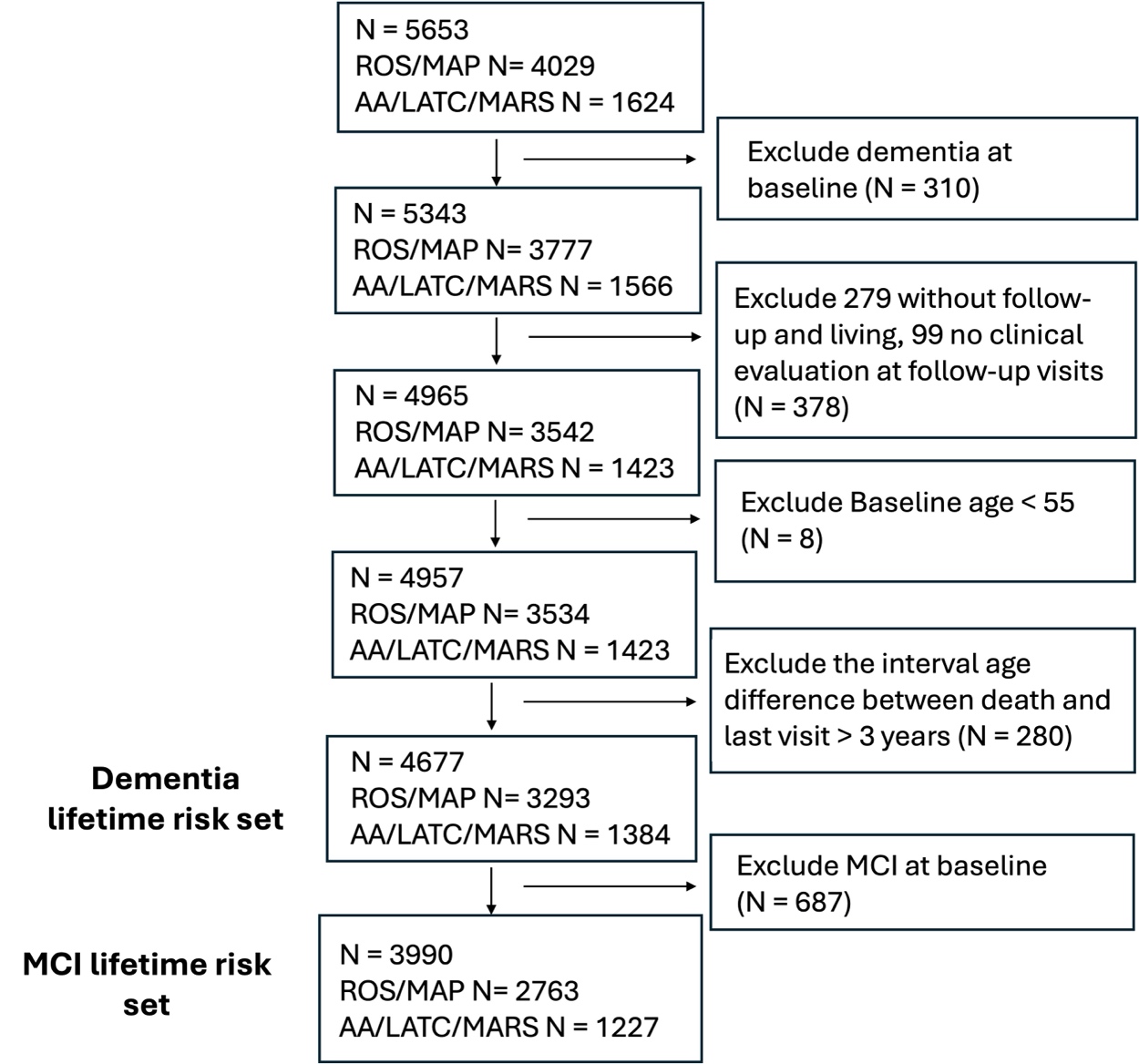


**Figure S1. STROBE Flow Diagram.**

Abbreviations: ROS, Religious Orders Study; MAP, Memory and Aging Project; MARS, Minority Aging Research Study; AACORE, Clinical Core; LATC, Latino Core; MCI, Mild Cognitive Impairment.


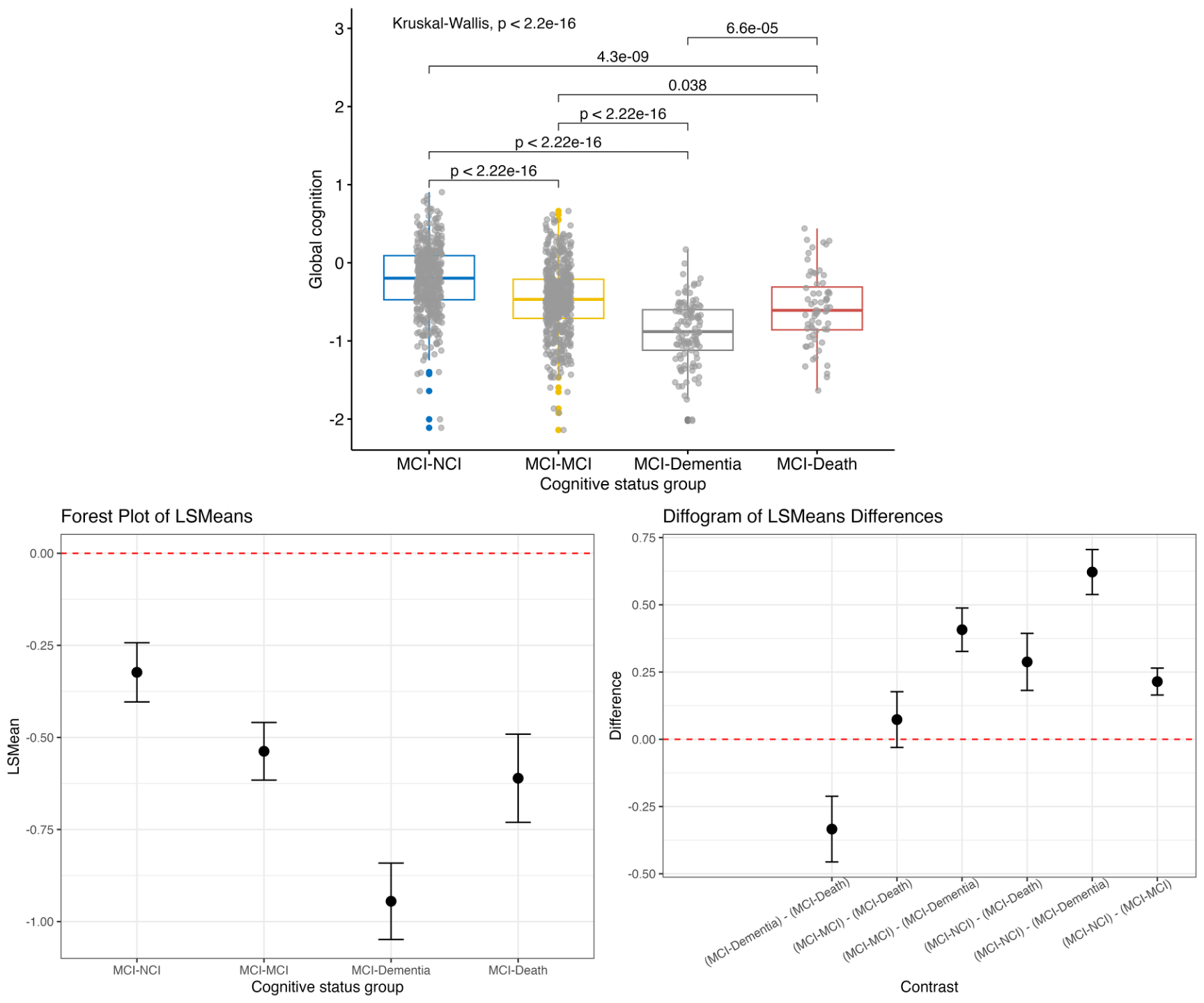


**Figure S2. Global Cognitive Performance Across Participant Subgroups Diagnosed with MCI at Baseline**

Top panel: Boxplot showing the distribution of baseline global cognitive scores across four mild cognitive impairment (MCI) subgroups based on clinical outcomes: MCI-NCI (n = 441; reverted to no cognitive impairment at the second visit), MCI-MCI (n = 559; consistent MCI), MCI-Dementia (n = 113; progressed to dementia at the second visit), and MCI-Death (n = 56; died after the first MCI diagnosis). The Kruskal-Wallis test revealed a significant overall group difference (p < 2.2 × 10⁻¹⁶). Pairwise post-hoc comparisons showed significant differences across all groups, except between MCI-MCI and MCI-Death (annotated p-values). Bottom left: Forest plot of least square means (LSMeans) of global cognitive scores for each cognitive status group, displaying estimated means and 95% confidence intervals from a linear model adjusted for age, sex, race & ethnicity and education. Bottom right: Diffogram illustrating pairwise LSMeans differences between groups, with 95% confidence intervals. Statistically significant differences were observed among all group comparisons except between MCI-MCI and MCI-Death.


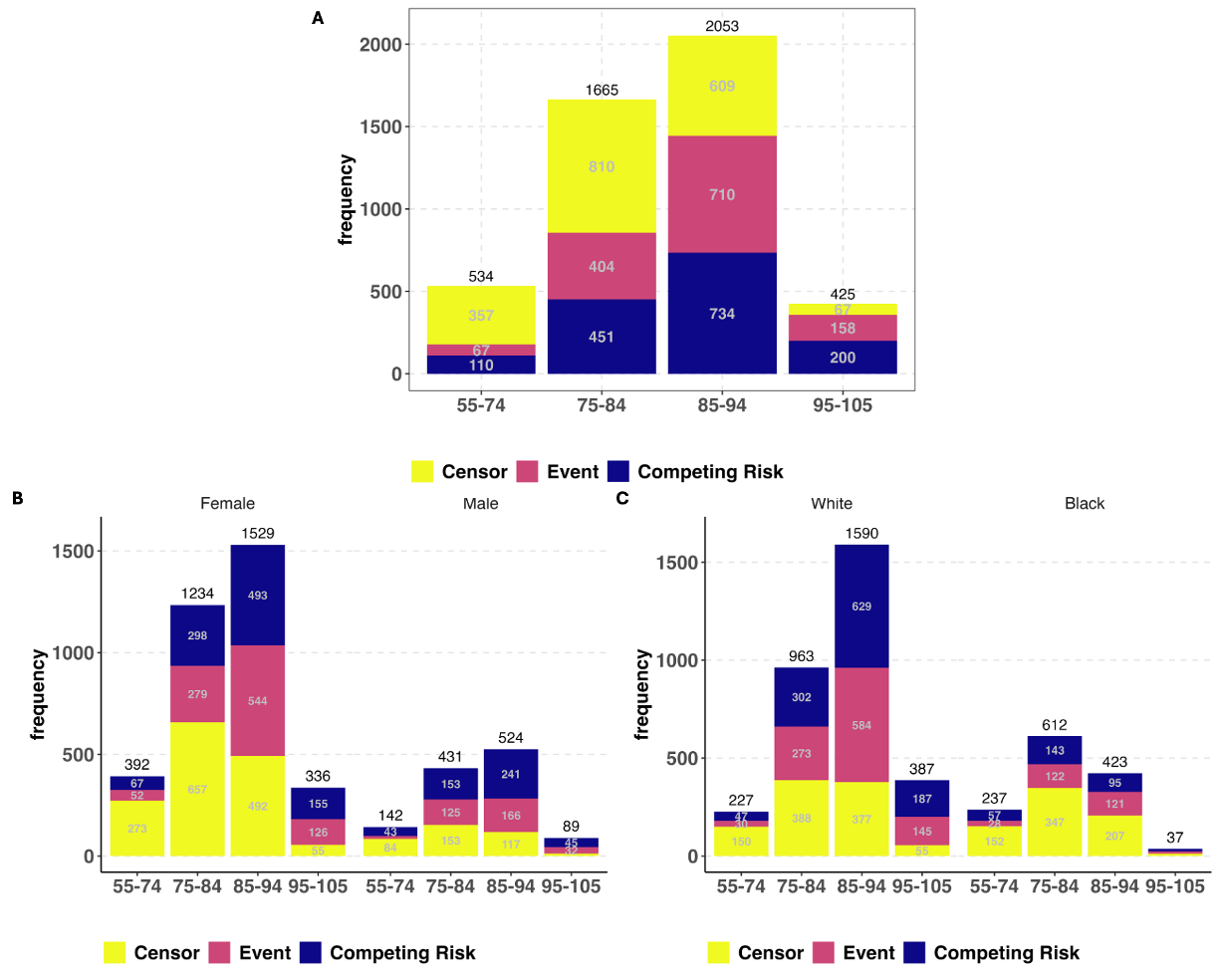


**Figure S3. Total number of participants in the dementia lifetime risk set per decade overall, by sex, and by race (n = 4611).** Panel A shows the overall distribution, Panel B shows the distribution by sex, and Panel C shows the distribution by race. The total number of participants (black text) at each age group is displayed on top of each bar. The numbers for each outcome (censor, event, and competing risk) (gray text) are displayed within each bar. *Censor* indicates participants who were alive without dementia at the end of follow-up. *Event* indicates participants who developed incident dementia. *Competing risk* indicates participants who died without developing dementia.


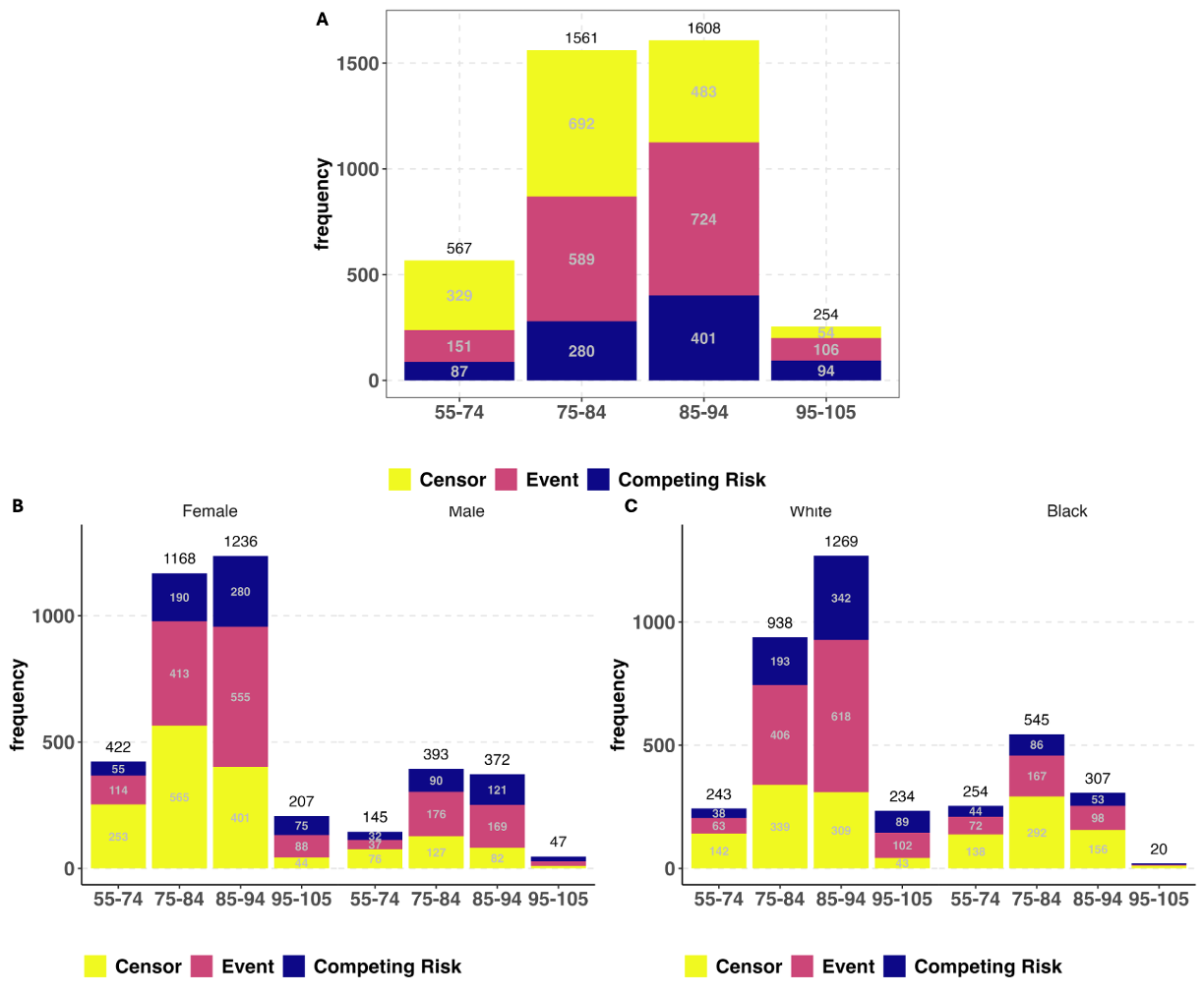


**Figure S4. Total number of participants in the MCI lifetime risk set per decade overall, by sex, and by race (n = 3915).** Panel A shows the overall distribution, Panel B shows the distribution by sex, and Panel C shows the distribution by race. The total number of participants (black text) at each age group is displayed on top of each bar. The numbers for each outcome (censor, event, and competing risk) (gray text) are displayed within each bar. *Censor* indicates participants who were alive without MCI at the end of follow-up. *Event* indicates participants who developed incident MCI. *Competing risk* indicates participants who died without developing MCI.


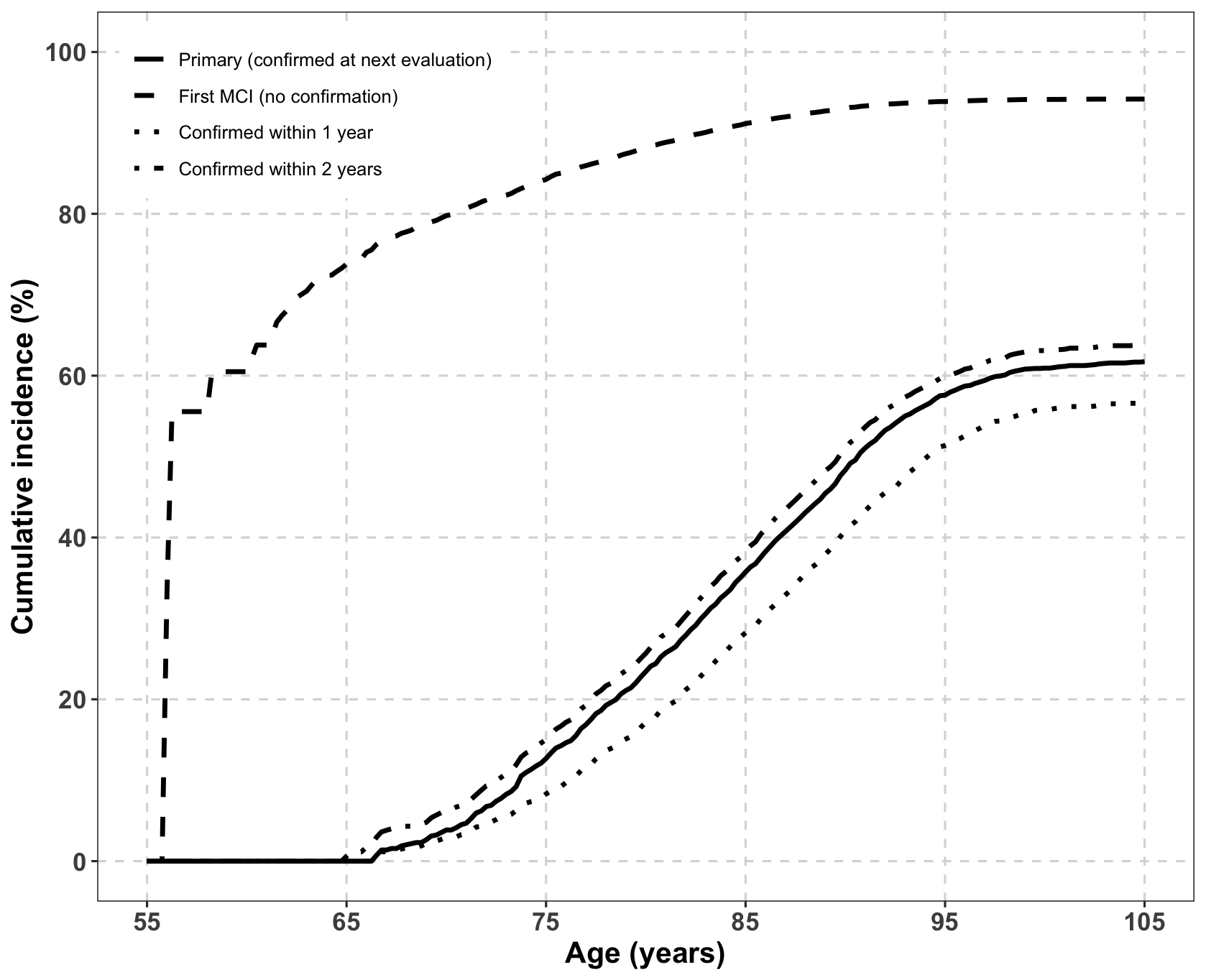


**Figure S5. Cumulative incidence of mild cognitive impairment (MCI) by age under alternative case definitions.** The solid line shows the primary definition, requiring confirmation at the next available evaluation or death. Dashed and dotted lines show sensitivity analyses defining MCI as the first clinical diagnosis without confirmation, or requiring confirmation within 1 year or 2 years, respectively. Less stringent definitions yield higher estimated lifetime risk and earlier apparent onset, whereas stricter confirmation windows produce lower estimates. Across definitions, the overall age-related pattern of cumulative incidence is preserved.

Abbreviation: MCI, Mild Cognitive Impairment.


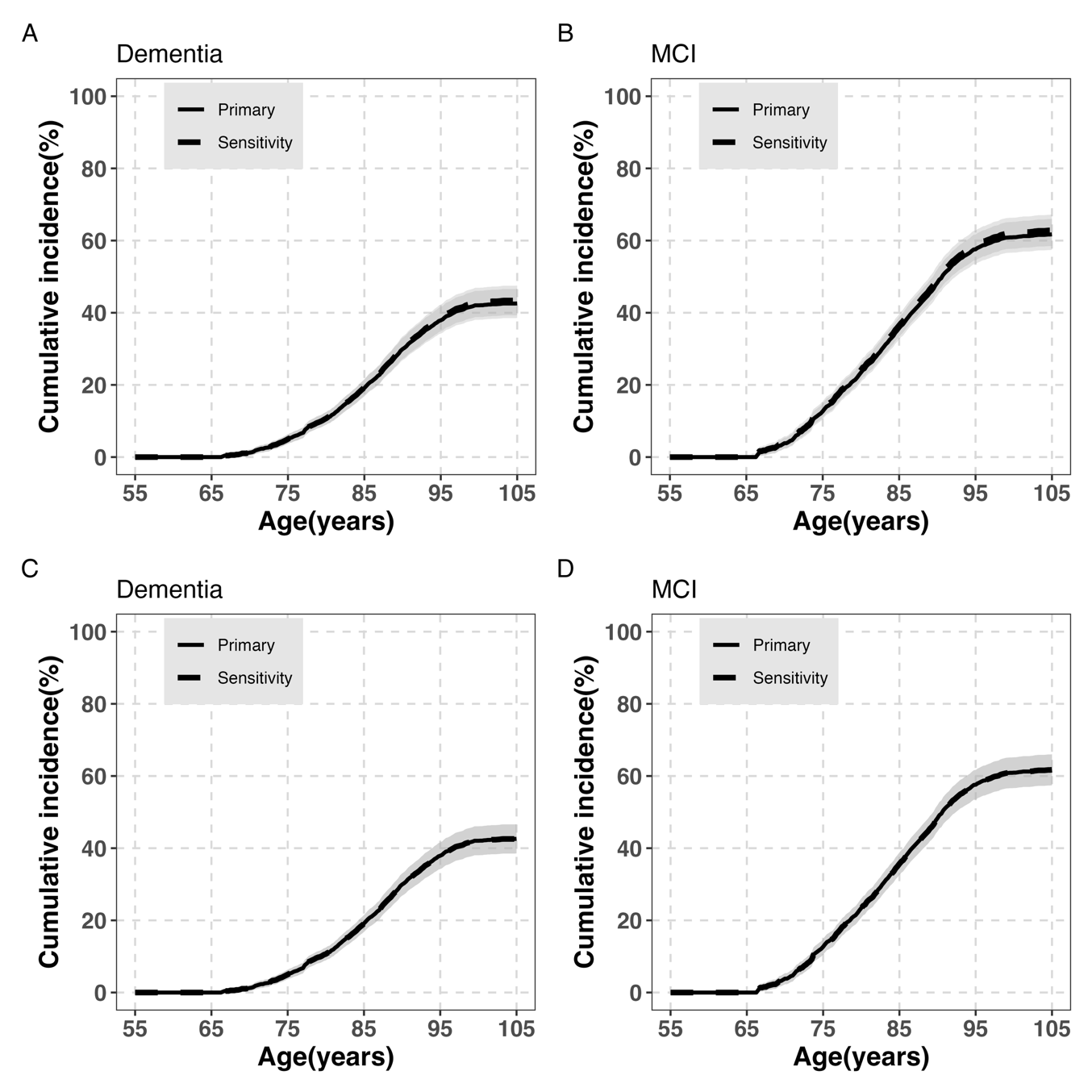


**Figure S6. Sensitivity analyses for lifetime risk of dementia and MCI under alternative handling of incomplete follow-up.** Panels A–B compare primary analyses excluding decedents whose last clinical evaluation occurred more than three years prior to death (solid lines) with sensitivity analyses retaining these individuals and censoring them at last contact (dashed lines). Panels C–D compare primary analyses excluding participants not yet eligible for an annual evaluation by the December 1, 2025 data cutoff with sensitivity analyses retaining these participants and administratively censoring them at last contact. In panels A-B, Lifetime risk curves for both dementia and MCI were nearly identical to those from the primary analysis across the age range. In Panels C–D, primary and administrative-censoring sensitivity curves overlap closely. Shaded areas indicate 95% confidence intervals.

Abbreviation: MCI, Mild Cognitive Impairment.


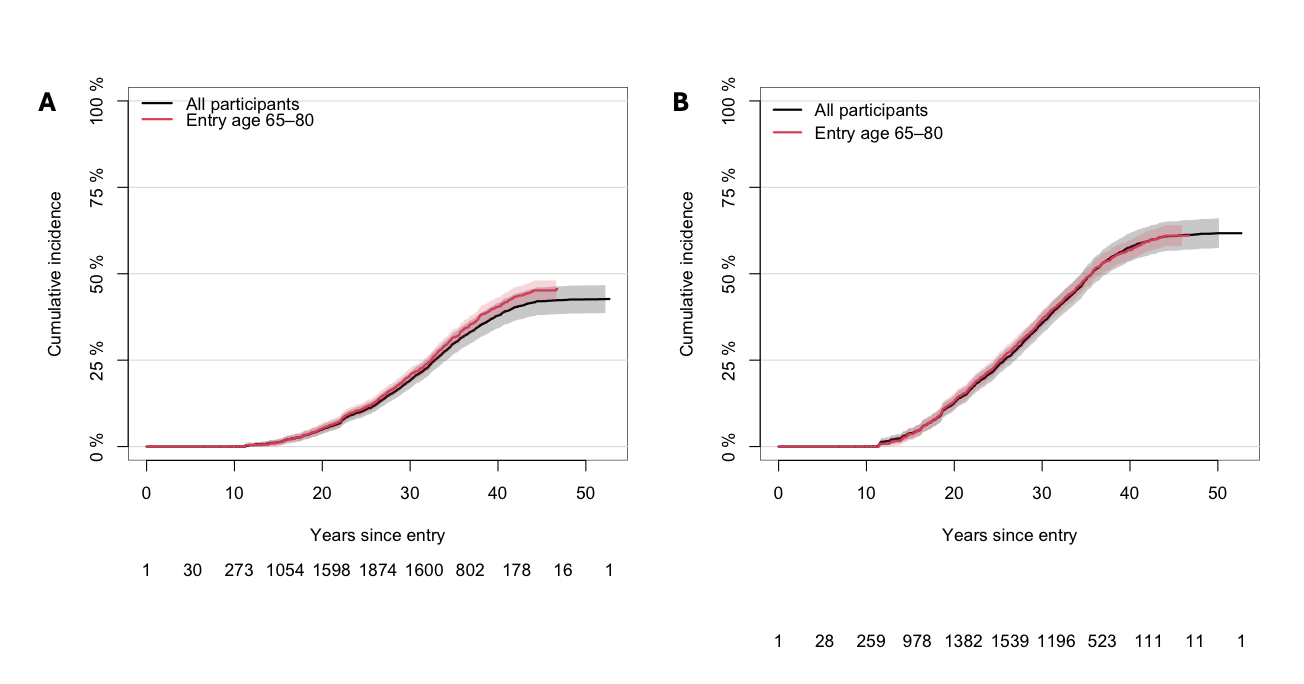


**Figure S7. Sensitivity of lifetime risk estimates to age at study entry.** Cumulative incidence of dementia/MCI estimated using the Aalen–Johansen estimator with age as the time scale and delayed entry. Estimates for the full analytic sample (black) and for participants entering between ages 65 and 80 (red) were similar across follow-up, indicating robustness of lifetime risk estimates to variation in age at cohort entry.


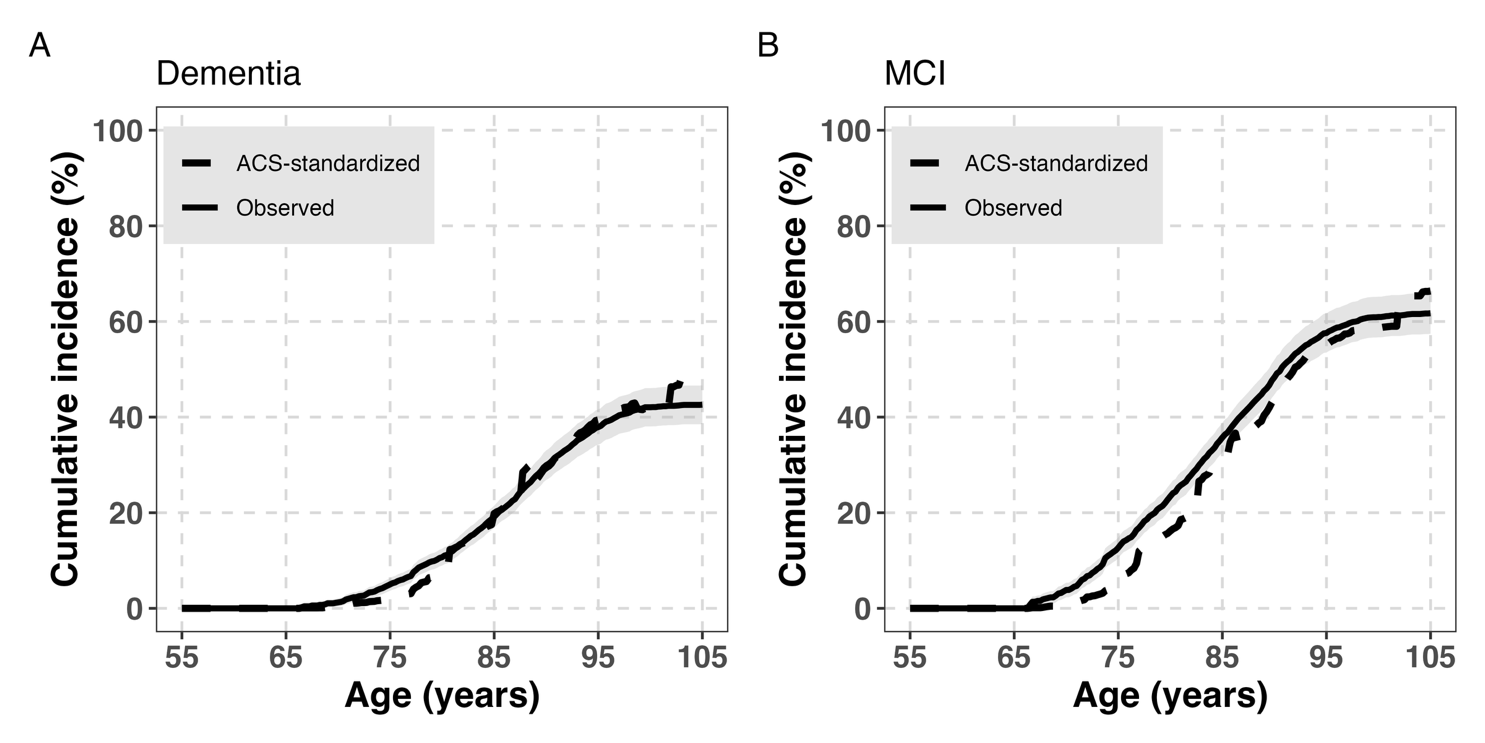


**Figure S8. Comparison of observed and ACS-standardized lifetime risk of dementia and MCI.** Cumulative incidence curves accounting for the competing risk of death are shown for dementia (left) and MCI (right). Solid lines represent observed estimates from the analytic sample; dashed lines represent estimates standardized to the 2019–2023 U.S. Census American Community Survey age × sex × race distribution. Shaded bands indicate 95% confidence intervals. Differences were most apparent at younger ages and attenuated at older ages, with overall trajectories remaining similar.

**
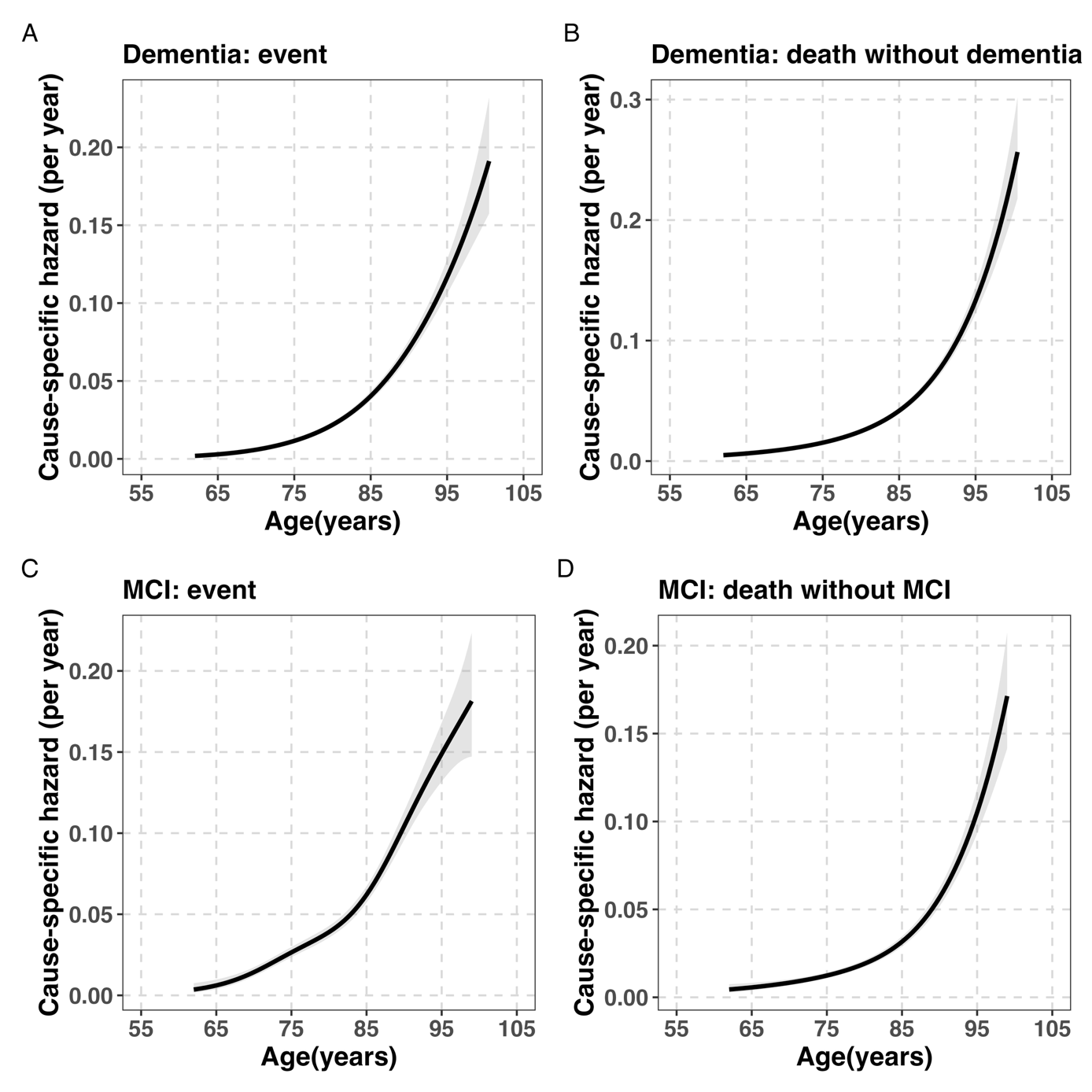
**

**Figure S9. Age-specific cause-specific hazards for dementia and MCI.** Smoothed cause-specific hazards (per year) estimated using generalized additive models with penalized splines and age as the time scale. Panels show hazards for (A) dementia, (B) death without dementia, (C) MCI, and (D) death without MCI. Shaded areas represent 95% confidence intervals. Although cumulative incidence curves plateau at advanced ages, cause-specific hazards continue to increase, particularly for competing mortality, consistent with selective survival and diminishing risk sets at extreme ages.


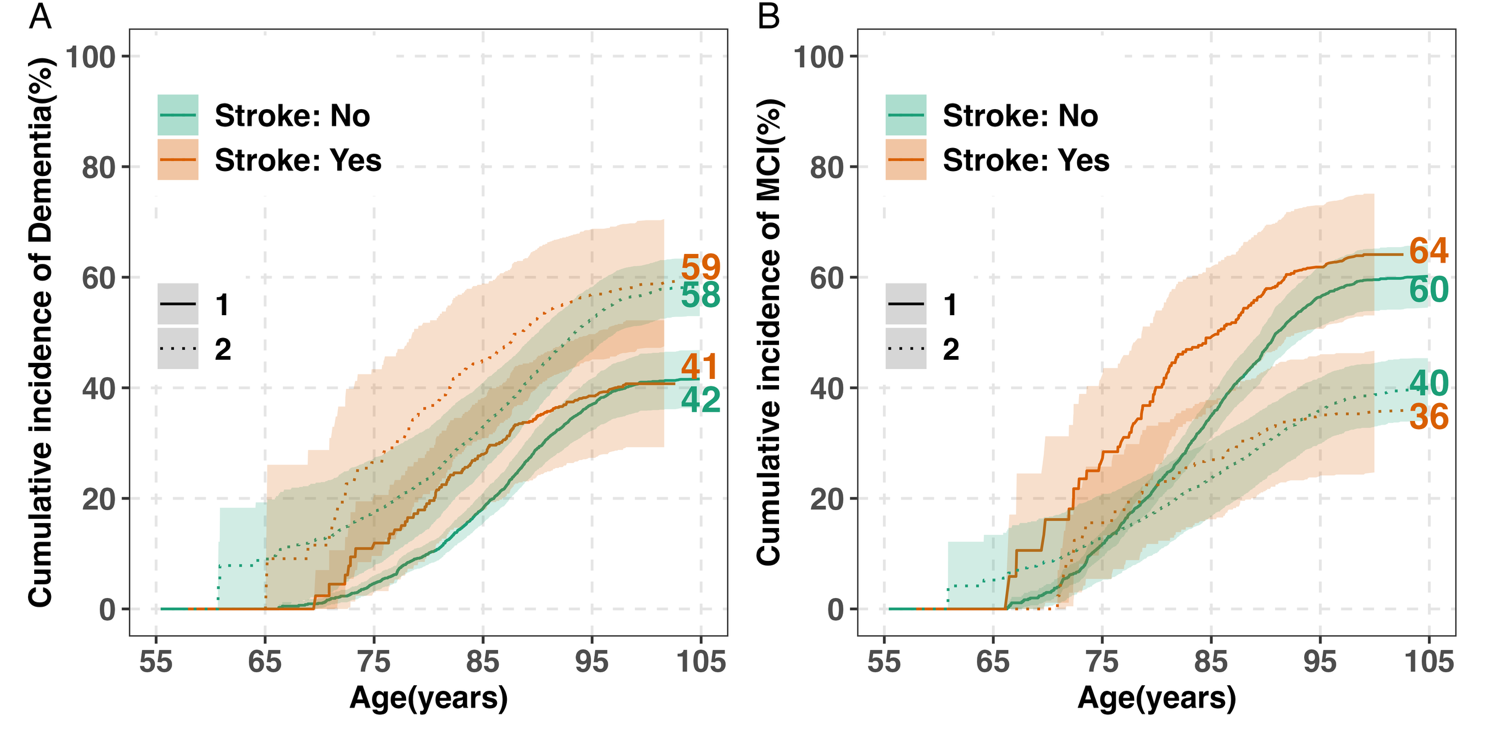


**Figure S10. Lifetime Risk of Dementia, MCI, and Death Without Diagnosis by Stroke History (Ages 55–105) in the Five RUSH Cohorts.** Panel A: The cumulative incidence of dementia (solid lines) and death without dementia (dashed lines) is shown for participants without and with stroke history at baseline (colored separately). Panel B: The cumulative incidence of MCI (solid lines) and death without MCI (dashed lines) is also shown for participants without and with stroke history at baseline. Estimates are percentages accounting for the competing risk of death. Shaded areas show 95% confidence intervals (CIs). Age-specific cumulative risks by stroke history are provided in Table S9 (dementia/MCI) and Table S10 (death without diagnosis).

Abbreviation: MCI, Mild Cognitive Impairment.


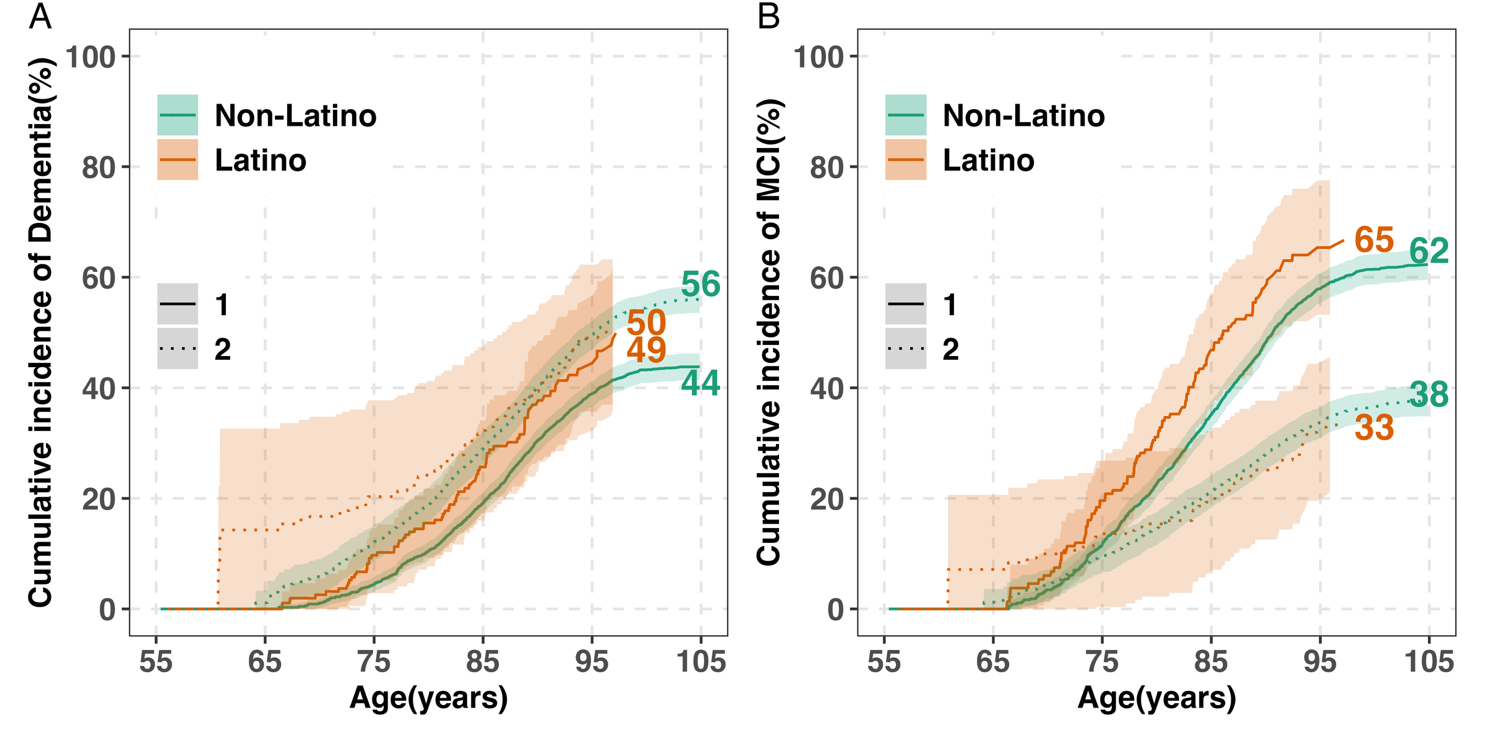


**Figure S11. Lifetime Risk of Dementia, MCI, and Death Without Diagnosis by Latino ethnicity (Ages 55–105) in the Five RUSH Cohorts.** Panel A: The cumulative incidence of dementia (solid lines) and death without dementia (dashed lines) is shown for non-Latino and Latino (colored separately). Panel B: The cumulative incidence of MCI (solid lines) and death without MCI (dashed lines) is also shown for non-Latino and Latino. Estimates are percentages accounting for the competing risk of death. Shaded areas show 95% confidence intervals (CIs). Age-specific cumulative risks by stroke history are provided in Table S9 (dementia/MCI) and Table S10 (death without diagnosis).

Abbreviation: MCI, Mild Cognitive Impairment.
